# Revolutionizing oncology: the role of Artificial Intelligence (AI) as an antibody design, and optimization tools

**DOI:** 10.1186/s40364-025-00764-4

**Published:** 2025-03-29

**Authors:** Varun Dewaker, Vivek Kumar Morya, Yoo Hee Kim, Sung Taek Park, Hyeong Su Kim, Young Ho Koh

**Affiliations:** 1 Institute of New Frontier Research Team, Hallym University, Chuncheon-Si, Gangwon-Do 24252 Republic of Korea; 2 Department of Orthopedic Surgery, Hallym University Dongtan Sacred Hospital, Hwaseong-Si, 18450 Republic of Korea; 3 Department of Biomedical Gerontology, Ilsong Institute of Life Science, Hallym University, Seoul, 07247 Republic of Korea; 4 Department of Obstetrics and Gynecology, Kangnam Sacred-Heart Hospital, Hallym University Medical Center, Hallym University College of Medicine, Seoul, 07441 Republic of Korea; 5 Department of Internal Medicine, Division of Hemato-Oncology, Kangnam Sacred-Heart Hospital, Hallym University Medical Center, Hallym University College of Medicine, Seoul, 07441 Republic of Korea; 6 EIONCELL Inc, Chuncheon-Si, 24252 Republic of Korea

**Keywords:** Cancer, Antibody, Artificial Intelligence, AI, ScFv, CAR-T cell, NK-cell therapy

## Abstract

Antibodies play a crucial role in defending the human body against diseases, including life-threatening conditions like cancer. They mediate immune responses against foreign antigens and, in some cases, self-antigens. Over time, antibody-based technologies have evolved from monoclonal antibodies (mAbs) to chimeric antigen receptor T cells (CAR-T cells), significantly impacting biotechnology, diagnostics, and therapeutics. Although these advancements have enhanced therapeutic interventions, the integration of artificial intelligence (AI) is revolutionizing antibody design and optimization. This review explores recent AI advancements, including large language models (LLMs), diffusion models, and generative AI-based applications, which have transformed antibody discovery by accelerating de novo generation, enhancing immune response precision, and optimizing therapeutic efficacy. Through advanced data analysis, AI enables the prediction and design of antibody sequences, 3D structures, complementarity-determining regions (CDRs), paratopes, epitopes, and antigen–antibody interactions. These AI-powered innovations address longstanding challenges in antibody development, significantly improving speed, specificity, and accuracy in therapeutic design. By integrating computational advancements with biomedical applications, AI is driving next-generation cancer therapies, transforming precision medicine, and enhancing patient outcomes.

**Graphical Abstract:**

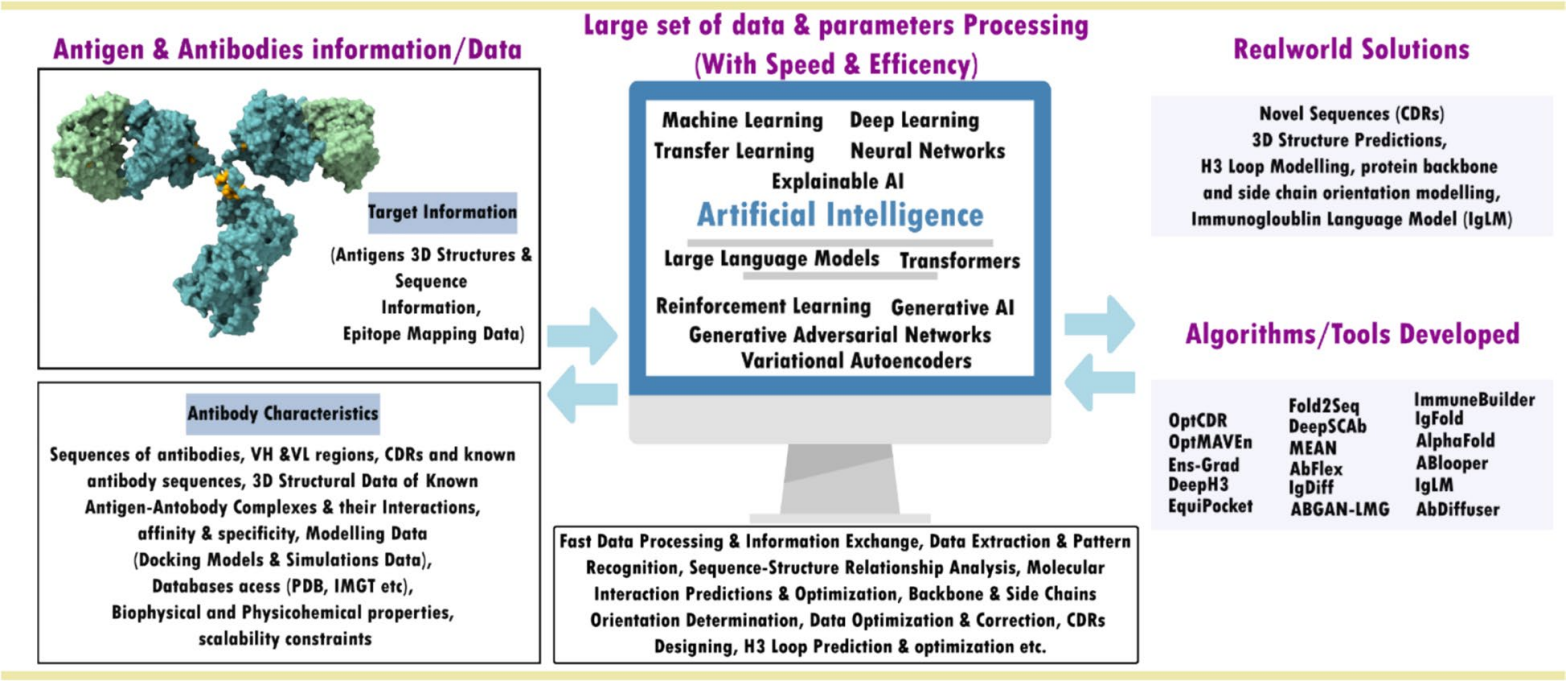

## Introduction

Antibody-based therapies have revolutionized oncology, with monoclonal antibodies (mAbs) becoming essential tools for targeted cancer treatment since their development in the late twentieth century [1]. These therapies selectively target antigens on malignant cells, minimizing damage to healthy tissues and improving treatment outcomes [1, 2]. However, tumor biology presents significant challenges due to cellular diversity, genetic mutations, and adaptive resistance mechanisms driven by both genetic and non-genetic factors [3, 4]. These complexities hinder the development of highly effective antibody-based treatments.

Genetic alterations in oncogenes and tumor suppressor genes drive malignancy, leading to rapid proliferation and resistance to apoptosis. Meanwhile, non-genetic factors, such as modifications in the tumor microenvironment and metabolic shifts, allow cancer cells to evade immune surveillance and adapt to therapy [3, 4]. Resistance mechanisms further complicate treatment; for example, hypoxia can reduce radiation effectiveness, while PI3K/AKT pathway mutations contribute to therapeutic resistance [3]. While targeted therapies, such as HER2 and VEGF inhibitors, have improved clinical outcomes, challenges like antigen heterogeneity, immune evasion, and immunosuppressive tumor microenvironments continue to limit their efficacy [3–8].

To address these challenges, researchers have developed advanced monoclonal antibody-based therapies, including bispecific antibodies (bsAbs), antibody–drug conjugates (ADCs), immune checkpoint inhibitors, chimeric antigen receptor (CAR)-T cells, and CAR-NK cells [9–11]. Despite their promise, these therapies face limitations such as off-target effects, drug resistance, stability issues, and immunogenicity. Additionally, tumor complexities such as inadequate chemokine trafficking, T-cell suppression, metabolic dysregulation, and high mutational burdens reduce the effectiveness of these treatments [12–16]. Immunosuppressive factors, including regulatory T-cells and myeloid-derived suppressor cells, further hinder immunotherapy, while resistance mechanisms, such as HER2-targeted drug resistance (HTDR), pose additional challenges [14, 17, 18]. Genetic factors, such as RAS mutations, can render therapies ineffective, whereas HER2 overexpression in breast cancer can influence treatment responses [14]. Thus, overcoming cellular complexity, genetic variability, and immune evasion remains critical for advancing antibody-based cancer therapies [14].

Early computational methods for antibody design were constrained by limited structural data and computational power, which hindered the development of reliable models for antibody-antigen interactions [19]. This led to inaccuracies in binding predictions, restricting their utility in guiding experimental design and necessitating extensive in vitro validation. As a result, researchers relied heavily on time-intensive and resource-intensive experimental approaches [19, 20]. However, recent advancements in high-throughput sequencing, the availability of structural data, and improved computational techniques have enabled more precise predictions of antibody-antigen structures and interactions [19, 21–26].

Artificial intelligence (AI), particularly machine learning (ML) and deep learning (DL), has transformed antibody design by improving the prediction of antibody-antigen structures, interactions, structural dynamics, and molecular stability [25, 26]. Leveraging vast structural databases like the Protein Data Bank (PDB) and advanced tools such as AlphaFold, AI-driven approaches enhance in-silico antibody design with exceptional efficiency and accuracy [27, 28]. With increased computational power and cloud-based platforms, these advancements enable rapid simulations that complement traditional experimental methods, improving antibody affinity, specificity, and therapeutic potential, particularly in cancer treatment [13, 29–33]. AI models analyze complex datasets to predict antibody sequences, 3D structures, complementarity-determining regions (CDRs), paratopes, epitopes, and antigen–antibody interactions with remarkable accuracy. These innovations streamline antibody design, optimization, and testing, reducing time and cost while addressing challenges related to developability and stability [26, 34–38].

This review explores AI’s transformative role in antibody design and optimization. We highlight advancements in CDR development, structural stability, folding efficiency, and CDR H3 conformation prediction—key factors in optimizing epitope-paratope interactions and enhancing therapeutic efficacy.

## Evolution of antibody-based therapeutics

**Fig. 1:**
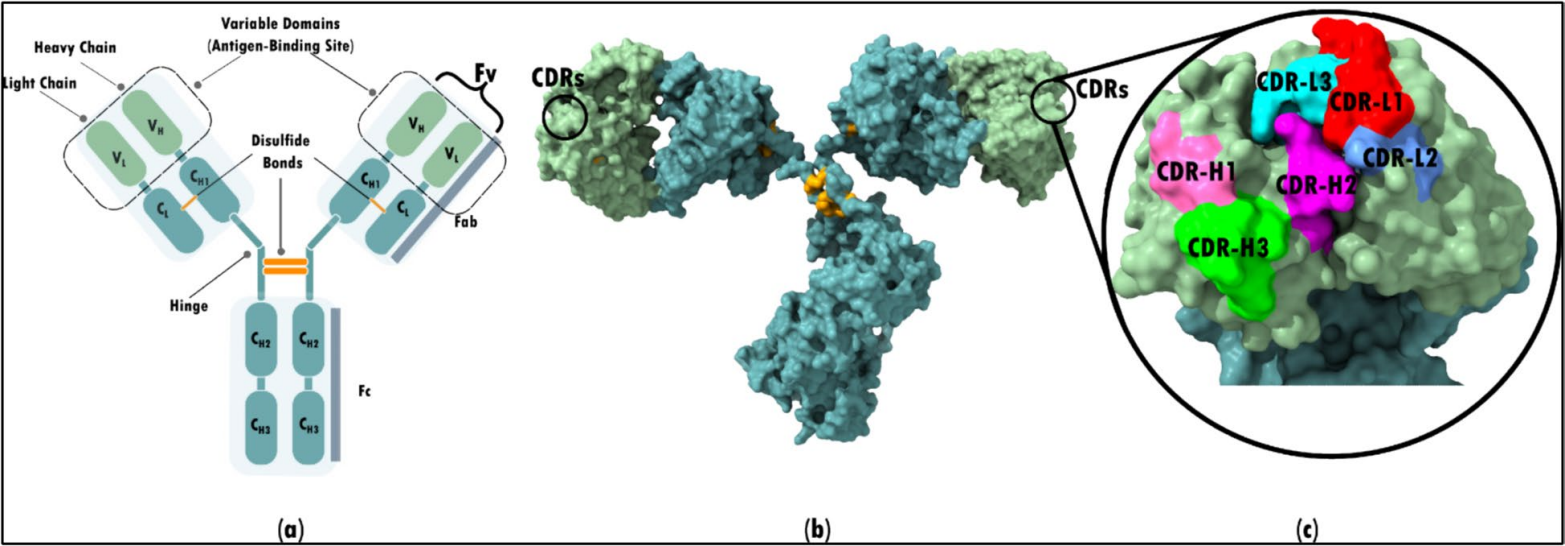
Overview of antibody structure. (**a**) The antibody has a Y-shaped structure, comprising two identical light chains (L) and heavy chains (H). These chains give rise to two Fab segments along with one Fc segment, comprising a total of 12 domains. The Fab segments are those where antigen recognition and binding occur, while the Fc region is primarily responsible for carrying out effector functions. (**b**) The surface representation of the antibody (PDB code: 1IGT) is depicted in Fig. 1(b). (**c**) Each Fab's antigen-binding site consists of six hypervariable loops: CDR-L1, CDR-L2, CDR-L3, CDR-H1, CDR-H2, and CDR-H3. These loops are situated on the variable domain pair VL: VH of each Fab, which is also referred to as the variable fragment (Fv)

Antibody-based therapies offer high specificity in cancer treatment by binding to antigens on cancer cells, triggering immune responses and inhibiting tumor growth with greater precision than traditional treatments [39, 40]. Advances in humanized antibody engineering, tumor biology, and antibody conjugation and selection techniques have further enhanced their efficacy [41]. These developments are closely tied to antibody structure, which consist of two light and two heavy chains forming a Y-shaped structure. The Fab regions mediate antigen binding, while the Fc region governs effector functions (Fig. 1) [42]. Structural modifications such as single-chain variable fragments (scFvs) and bispecific antibodies (bsAbs) enable targeting of multiple antigens, further improving therapeutic effectiveness [43]. Notably, an antibody's specific binding capability depends upon its six hypervariable regions: CDR-H1, H2, H3 (heavy chain), and CDR-L1, L2, and L3 (light chain) [44, 45].

Currently, over 100 mAb drug are approved, with many more in clinical trials or patent filing stage [34, 46]. A total of 162 antibody therapies have been approved globally, including 122 in the US, 82 in Japan, 114 in Europe, and 73 in China [47]. The projected revenue for antibody therapies is estimated to reach $479 billion by 2028. (www.marketsandmarkets.com).

First approved in the 1980s, mAbs paved its way for advanced therapies, including bsAbs, ADCs, CAR T-cell and CAR NK-cell therapies, and immune checkpoint inhibitors. Most prominent examples of mAbs include rituximab (Rituxan) for non-Hodgkin lymphoma and chronic lymphocytic leukemia, targeting CD20 on B cells [48]; trastuzumab (Herceptin) for HER2-positive breast cancer [49]; cetuximab (Erbitux) for colorectal, head, and neck cancers, targeting EGFR [49]; bevacizumab (Avastin) for various cancers by blocking VEGF and inhibiting tumor angiogenesis [49]; and ipilimumab (Yervoy) for melanoma, targeting CTLA-4 to enhance immune response [50]. Combining these mAbs with chemotherapy has shown improved outcomes [40, 51].

**Fig. 2:**
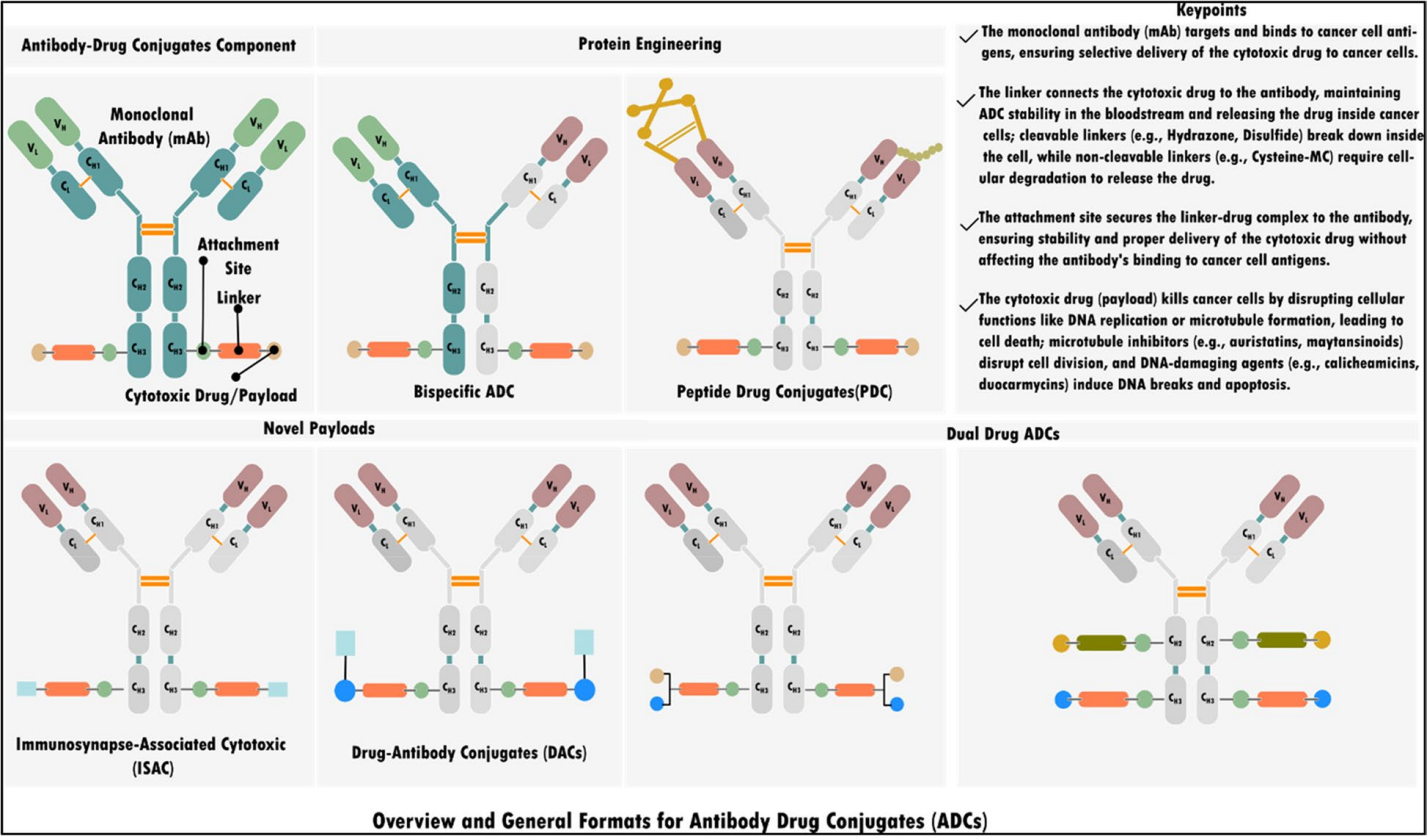
Overview of ADC components and their general formats. The diagram illustrates each component: the mAb, attachment site, linker, and cytotoxic drug/payload. These components work together to selectively target and kill tumor cells, leveraging the specificity of mAbs and potency of cytotoxic drugs

ADCs, also known as "biological missiles," are a breakthrough in targeted cancer therapy by combining the specificity of mAbs with potent cytotoxic drugs [51, 52]. ADCs consist of a tumor-specific mAb, a cytotoxic drug, and a linker, ensuring stability in circulation and targeted drug release within cancer cells (Fig. 2) [51]. This structure enhances precision, sparing healthy tissues and offering higher clinical response rates than unconjugated mAbs targeting the same surface antigens [53]. Advances in linker technology and potent cytotoxic payloads further improved ADC efficacy and safety [54]. Most prominent ADCs include brentuximab vedotin, effective for Hodgkin's lymphoma and systemic anaplastic large cell lymphoma [53], and ado-trastuzumab emtansine, approved by the FDA for HER2-positive breast cancer [55].

**Fig. 3:**
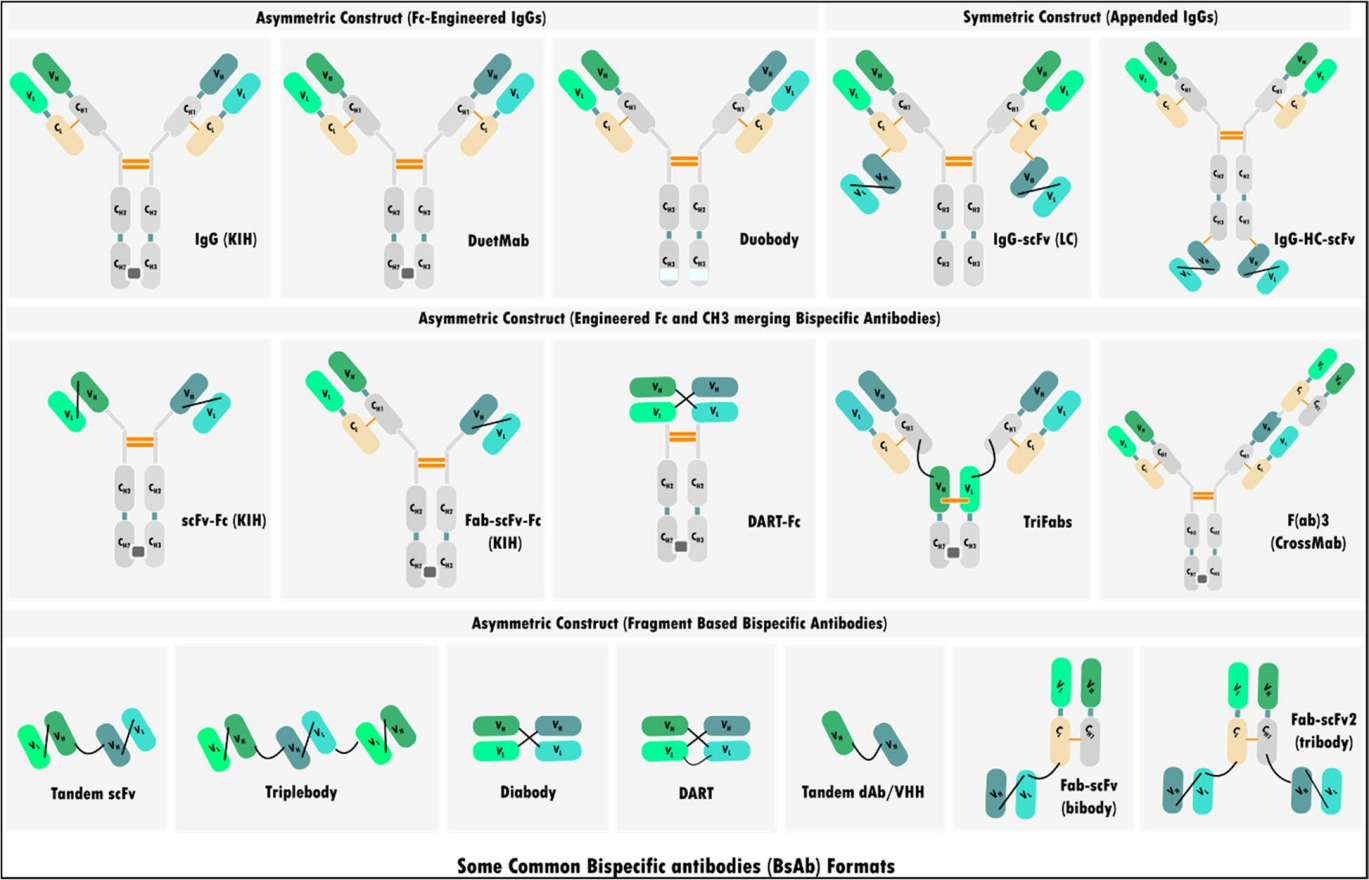
The figure shows various common bispecific antibody (bsAb) formats. The Fc-modified IgG format leverages KIH technology to facilitate the heterodimerization of two distinct heavy chains. To enhance the pairing of homologous heavy and light chains, DuetMab introduces an alternative disulfide bond, replacing the natural bond at one CH1-CL interface. The Duobody format includes specific Fc region mutations, significantly reducing Fc-mediated cytotoxicity. Appended IgG structures integrate an IgG with a single-chain variable fragment (scFv), either through light chain (LC) or heavy chain (HC) connections. Constructs such as scFv-Fc and Fab-scFv-Fc also rely on the KIH method for their assembly. The DART-Fc structure incorporates two distinct antigen-binding domains, stabilized into a diabetes-like mimic. TriFabs are IgG-derived bsAbs with two standard Fab arms linked to a third Fab-sized unit via flexible peptide linkers. CrossMab, on the other hand, achieves connectivity using domain crossovers involving a shared light chain. Tandem scFv (taFv) represents the most compact bsAb design, closely related to Triplebody constructs. The diabetic (db) format employs a short linker to join VH and VL domains of an scFv, forming a noncovalent heterodimer. Dual-Affinity Re-Targeting (DART) molecules pair two Fv segments to generate distinct antigen-binding regions. Tandem single-domain antibodies (dAb/VHH) are derived from the binding regions of heavy-chain-only antibodies. Lastly, the Fab-scFv "bibody" format links an scFv to the Fab's C-terminus, while the Fab-scFv "tribody" format adds a second scFv segment for enhanced functionality

bsAbs, such as blinatumomab (for acute lymphoblastic leukemia) and catumaxomab (for malignant ascites), have revolutionized cancer therapy by targeting two antigens simultaneously, offering increased tumor selectivity and potential for improved payload delivery (Fig. 3) [56]. Also Amivantamab, targeting EGFR and MET (Mesenchymal-Epithelial Transition factor), has shown promising results in treating NSCLC with EGFR exon 20 insertion mutations [57]. These therapies can target multiple surface receptors or ligands linked to cancer, growth, or inflammation [43]. They can also prevent cancer cell escape mechanism by blocking multiple pathways [58]. Furthermore, bsAbs have shown promise in treating both blood cancers and solid tumors [56], but more research is required to optimize their use and minimize toxicity.

**Fig. 4:**
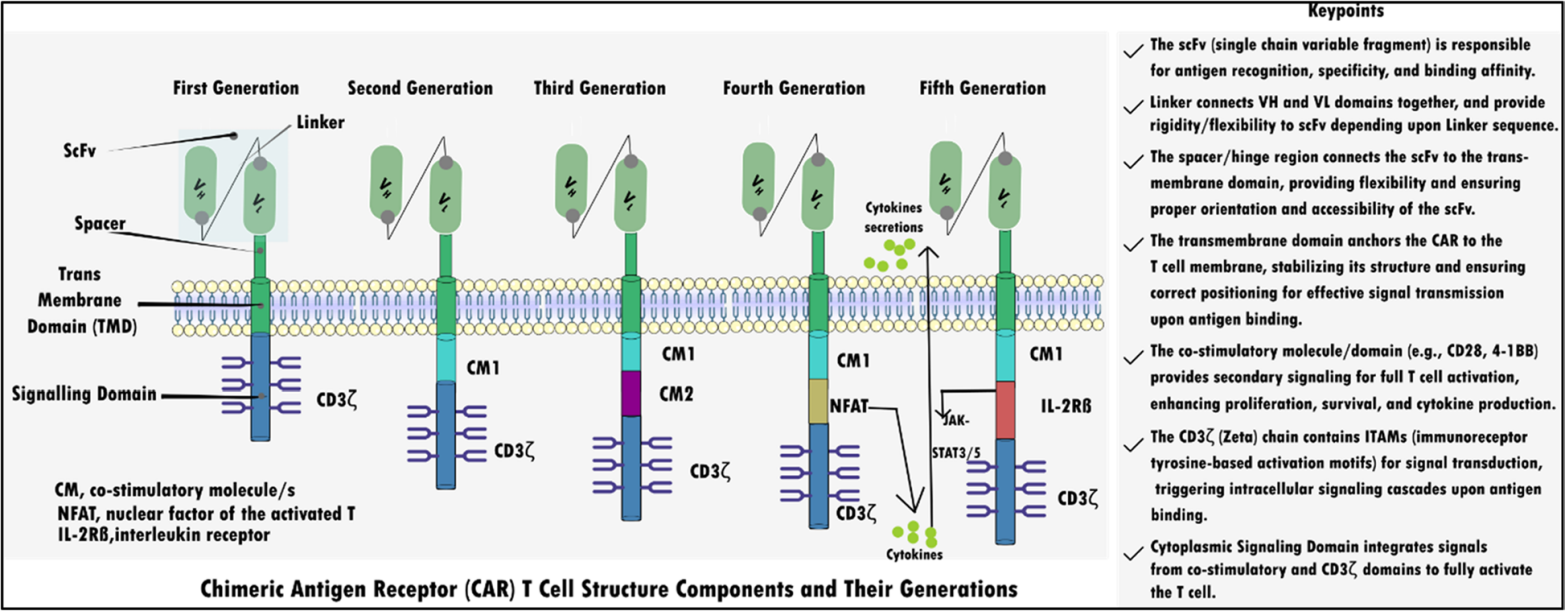
Schematic representation of the evolution of the CAR structure from the first generation to the fifth. First CAR generation contains only a CD3ζ signaling domain and no co-stimulatory molecules (CMs). Second generation CAR adds one CM to CD3ζ, enabling dual signaling. Third generation CAR combines CD3ζ with multiple CMs to enhance signaling. The fourth-generation CAR, like the 2G CAR, features an NFAT-responsive cassette that triggers cytokine expression, delivering triple signaling through CD3ζ, CM, and transgenic proteins. The fifth generation of CAR, rely on 2G CAR and integrates IL-2Rβ receptors, which activates the JAK-STAT signaling domain for synergistic activation of CD3ζ, CMs, and the JAK-STAT3/5 pathway

CAR T-cell therapy (Fig. 4) has transformed cancer treatment, particularly in blood cancers, with FDA-approved treatments like tisagenlecleucel for acute lymphoblastic leukemia, axicabtagene ciloleucel for non-Hodgkin lymphoma, and brexucabtagene autoleucel for mantle cell lymphoma [59–61]. Other therapies, including idecabtagene vicleucel, lisocabtagene maraleucel, and ciltacabtagene autoleucel, are also used for specific adult cancers [62–65]. Idecabtagene vicleucel treats relapsed multiple myeloma [63], lisocabtagene maraleucel targets relapsed large B-cell lymphoma [64], and ciltacabtagene autoleucel is used for relapsed/refractory multiple myeloma [65]. This personalized immunotherapy engineers a patient's T cells to attack cancer cells, showing significant success in leukemia and lymphoma, receiving FDA approval [66]. However, challenges like high costs and severe side effects remain [67]. Further research is needed to extend CAR T-cell therapy to solid tumors and enhance its safety and effectiveness [66, 67].

CAR-NK cell therapy shows promise in cancer treatment by harnessing natural killer cells' innate immunity [68, 69]. It offers benefits such as reduced risks of Cytokine Release Syndrome, neurotoxicity as well as Graft-versus-Host Disease etc. [69]. Early trials, including a Phase I/II study targeting CD19 in B-cell lymphomas and leukemia, have yielded encouraging results [69]. CAR-NK cells also target solid tumor antigens such as HER2, EGFR and mesothelin, showing effective tumor infiltration and anti-tumor responses [70]. Challenges include ensuring long-term persistence and overcoming the immunosuppressive tumor microenvironment [71]. Despite these hurdles, CAR-NK cell therapy has significant potential as an innovative cancer treatment.

Immune checkpoint inhibitors targeting CTLA-4 and PD-1 have revolutionized cancer therapy by enhancing the immune system's capacity to combat tumors. Inhibitors such as Ipilimumab, Pembrolizumab, Nivolumab, Atezolizumab, and Durvalumab have achieved significant success in treating cancers such as melanoma, lung, kidney, and bladder, improving patient survival rates [72–75]. These inhibitors work by disrupting proteins that help cancer cells evade detection, thereby empowering the immune system to target and destroy them. However, not all patient responds hence emphasizing the need for predictive biomarkers [74]. While they are effective, these drugs can cause side effects like fatigue, rashes, fever, and, in rare cases, serious issue like cardiotoxicity and inflammation [72].

The choice between these innovative therapies requires a nuanced evaluation of their benefits and limitations, with decisions tailored to each patient's unique clinical needs. Despite the precision of mAbs and ADCs, the novel mechanisms of bsAbs and CAR-T therapy, or the broad efficacy of immune checkpoint inhibitors, cancer treatment is advancing toward more personalized, effective, and less toxic options.

While these advancements have revolutionized treatment, the development of novel antibodies remains a complex challenge. Conventional antibody design faces significant obstacles, including the vast combinatorial search space of CDR sequences, off-target effects, low binding affinity, stability issues, and developability limitations such as poor expression, solubility, and aggregation [76–79]. Experimental discovery methods like phage display and rational design, though valuable, are time-intensive, labor-intensive, and impractical for exploring the immense diversity of potential antibody sequences [35]. Biophysical energy-based computational approaches enhance efficiency but remain computationally expensive and susceptible to local optima, limiting their ability to comprehensively explore sequence space [80–83].

AI, particularly ML and DL, is transforming antibody design by accelerating discovery and optimization while addressing key limitations of traditional methods [35–37, 84]. Generative models, such as generative adversarial networks (GANs) and variational autoencoders (VAEs), are now capable of generating diverse CDR sequences while improving binding affinity and stability properties [22, 85]. Reinforcement learning (RL) further optimizes antibody sequences by iteratively refining affinity and developability through feedback-driven improvements [35, 36, 86, 87]. Additionally, DL models such as convolutional neural networks (CNNs) and recurrent neural networks (RNNs) integrate sequence and structural data to predict how CDR modifications influence binding and stability, enabling more informed design decisions [88]. AI-powered simulations of antibody-antigen interactions further enhance target specificity and reduce off-target effects, addressing concerns related to immunogenicity [35, 36]. Furthermore, AI-driven high-throughput screening is expediting candidate selection, significantly reducing the time and cost associated with experimental validation [35, 36]. By efficiently navigating vast sequence spaces, optimizing antibody structures, and streamlining the development process, AI is playing an increasingly critical role in therapeutic antibody engineering. These innovations have the potential to improve the safety, efficacy, and manufacturability of antibody-based treatments. In the following section, we examine the foundational contributions of early ML approaches to antibody design and optimization, setting the stage for more advanced AI-driven innovations.

## Early ML approaches in developability of antibody design and optimization

Antibodies are vital therapeutics, but factors such as solubility, stability, and aggregation, along with developability attributes like viscosity, immunogenicity, and expression yield, play a critical role in ensuring their safety, scalability, and efficacy [9, 89]. Therefore, early-stage assessments using computational methods are essential for optimizing antibody candidates, minimizing risks, and ensuring efficient development [9, 89]. Various algorithms, including Support Vector Machines (SVMs), XGBoost, Random Forest, Gradient Boosting Machine (GBM), and k-nearest neighbors (k-NN), as well as DL models such as RNNs, CNNs, and Feedforward Neural Networks (FNNs), are widely used for predicting physicochemical and developability properties. Additionally, specialized methods like Repeated Edited Nearest Neighbor (RENN) and Instance Hardness Threshold (IHT) contribute to epitope prediction, structure modeling, and antigen–antibody interaction analysis are discussed in the following sections.

### Developability

Developability is a key factor in advancing antibody candidates into therapeutic products [9, 90]. It involves evaluating biophysical, biochemical, pharmacological, and manufacturability attributes to predict stability, efficacy, and safety. Biophysical properties such as stability, solubility, and aggregation propensity influence formulation stability and storage conditions, ensuring long-term viability [11, 90]. Solubility is particularly important, as poor solubility can hinder formulation and delivery. Biochemical factors, including Fc receptor interactions and post-translational modifications (e.g., glycosylation), directly impact function and pharmacokinetics, affecting antibody performance in vivo [9]. Pharmacological considerations, such as efficacy, safety, and off-target effects, determine therapeutic potential and risk profiles [10, 90]. Additionally, manufacturability aspects, including yield, purity, scalability, and cost-effectiveness, are essential for large-scale production and commercial viability [11].

An important aspect of developability is its relationship to immunogenicity, which influences therapeutic success. While developability focuses on manufacturability, stability, and function, immunogenicity assesses the likelihood of an antibody triggering an immune response. Although not always classified within developability, immunogenicity remains a crucial consideration, as highly immunogenic antibodies may require further optimization. To address these factors, high-throughput screening assays and computational tools play a pivotal role in modern antibody development. Screening assays rapidly evaluate thousands of variants for key properties like binding affinity, stability, and solubility, facilitating the selection of promising candidates [10, 11]. Meanwhile, computational tools leveraging ML and structural modeling predict risks such as aggregation and immunogenicity, allowing for early intervention [11]. Integrating these approaches streamlines development, minimizes failure risks, and enhances therapeutic antibody design efficiency [9, 10].

ML methods such as SVMs and Multilayer Perceptrons have demonstrated high accuracy in early-stage antibody screening and developability optimization, as shown in studies using datasets of up to 2,400 antibodies [91]. Algorithms such as XGBoost and PyCaret modeled IgG developability using biophysical properties, leveraging 250,000 models to predict hydrophobic patch energies and CDR charges [90]. A Random Forest model trained on 64 monoclonal antibodies (mAbs) identified correlations between biophysical properties and pharmacokinetics. The study found that poly-specificity and isoelectric point (pI) contributed to slower clearance rates, whereas hydrophobicity and extreme charges were linked to faster clearance. This approach supports early pharmacokinetic profiling, aiding in antibody design and reducing the risk of clinical trial failures [92]. Additionally, structure-based approaches predict critical properties such as pI, viscosity, and clearance, emphasizing the relationships between charge and stability, as well as surface charge and viscosity, to optimize high-concentration antibody formulations [92, 93]. Hu-mAb, an ML-based tool for antibody humanization, evaluates "humanness" scores and recommends mutations to minimize immunogenicity while maintaining functionality, validated using a dataset of 481 antibodies [94]. BioPhi, an open-source DL platform, integrates Sapiens for humanization and OASis for humanness scoring. Tested on 177 antibodies, it demonstrated expert-level accuracy in distinguishing human from non-human sequences. With both web and command-line access, BioPhi simplifies antibody development [95]. These innovations harness natural antibody diversity and ML to develop safer, more effective therapies.

### Solubility

Solubility is a major crucial factor in antibody design, directly influencing formulation, manufacturability, stability, and pharmacokinetics. High solubility is essential to prevent aggregation, ensure consistent dosing, and facilitate large-scale production. Poor solubility complicates purification, storage, and scalability, increasing costs and raising immunogenicity risks. Additionally, solubility affects pharmacokinetics by influencing absorption, distribution, and bioavailability [89, 96]. Key factors affecting solubility include amino acid composition, surface properties, post-translational modifications, and environmental conditions. Hydrophobic residues promote aggregation, whereas charged residues enhance solubility. In addition to sequence composition, glycosylation stabilizes protein folding, while formulation factors such as pH and excipients help maintain solubility. Addressing these factors through various strategies can enhance solubility and stability [97, 98]. Several approaches improve solubility, including sequence optimization to reduce hydrophobicity, glycoengineering, formulation adjustments with stabilizing excipients, structural modifications, and high-throughput screening to identify candidates with superior solubility profiles [98, 99].

To complement these experimental approaches, AI-driven predictive models have been developed to assess and enhance antibody solubility early in the design process. For instance, SOLpro is a sequence-based tool for predicting protein solubility during overexpression, utilizing 23 feature groups and a two-stage SVM model trained on over 17,000 proteins. It achieves 74% accuracy in tenfold cross-validation, outperforming models like PROSO in detecting insoluble proteins, and supports experimental planning and mutation design for improved solubility in protein engineering [100]. Similarly, CamSol and FoldX predict antibody solubility and stability through phylogenetic filtering. Validated on six antibodies, including nanobodies and scFv fragments, these models improved solubility and stability across 42 designs without compromising binding functionality [89, 96, 101].

Further advancements include PaRSnIP, which employs Gradient Boosting Machine algorithms to predict solubility with 74.11% accuracy—outperforming SOLpro and PROSO II by 9%. By integrating sequence features such as peptide frequencies with structural attributes, it identifies solubility determinants, including exposed residue thresholds and tripeptides like IHH [102]. SOLart, a Random Forest-based model, predicts protein solubility while minimizing aggregation risks, achieving a Pearson correlation of ~ 0.7 with experimental data [99]. Additionally, solPredict specializes in antibody solubility assessments for high-concentration monoclonal antibody (mAb) formulations. Unlike conventional models, it utilizes ESM1b embeddings without 3D modeling, demonstrating strong correlations with experimental solubility data across 260 antibodies. This tool enhances early-stage candidate selection by identifying solubility risks before experimental validation [103].

### Aggregation and viscosity

Aggregation and viscosity are key biophysical properties in antibody design, influencing developability, manufacturability, and therapeutic efficacy [9, 104]. Aggregation occurs when antibodies self-associate due to hydrophobic or electrostatic interactions, environmental stressors, or post-translational modifications. This leads to reduced efficacy, increased immunogenicity, and manufacturing challenges [9, 11, 104, 105]. Additionally, aggregation can increase viscosity, further complicating formulation and administration [9, 10, 106]. Strategies to minimize aggregation include sequence optimization to eliminate aggregation-prone motifs, formulation adjustments with stabilizing excipients, glycoengineering to enhance stability, and high-throughput screening for low-aggregation variants [9, 11, 104, 106].

Viscosity, a critical factor in high-concentration antibody formulations, affects subcutaneous injection, manufacturing, and product stability [9, 104, 106]. It arises from charge-charge or hydrophobic interactions [9]. Mitigation strategies include charge engineering to optimize charge distribution, sequence and structural modifications to reduce intermolecular interactions, and excipients to lower viscosity [9, 11]. Optimizing both aggregation and viscosity enhances bioavailability, ease of administration, and regulatory compliance, ultimately improving clinical success [9, 11, 107]. To facilitate this process, computational tools have been developed to predict and optimize these properties in antibody formulations.

Aggrescan3D (A3D) 2.0 predicts and enhances protein solubility by identifying aggregation-prone regions. It integrates CABS-flex for flexibility simulations, an automated mutation tool for reducing aggregation, and FoldX for stability assessments to maintain structural integrity. This approach improves solubility without compromising functionality and has been validated across various proteins [108]. In therapeutic mAbs, solution viscosity is critical for high-concentration formulations. Studies have identified net charge and Fv amino acid regions as key factors, with hydrophilic profiles linked to high viscosity. The High Viscosity Index (HVI) has been introduced as a rapid screening tool for identifying high-viscosity candidates. ML models, including logistic regression and decision trees, were validated on 27 FDA-approved mAbs, demonstrating high accuracy in viscosity classification [109]. Additionally, a k-nearest neighbors (KNN) model showed strong correlations (r = 0.89) for predicting aggregation rates and viscosity in high-concentration formulations, using features such as CDRH2 positive charge and hydrophobic surface area [110]. These computational models enable efficient screening and support the development of stable, high-concentration formulations for clinical applications.

### Epitope prediction

Epitope prediction is a crucial aspect of antibody design. It helps identify antigen regions targeted by antibodies for vaccine development, therapeutics, and diagnostics [111]. It plays a pivotal role in designing antibodies with high specificity and minimal off-target effects. In vaccine development, it helps identify immunogenic regions that elicit strong immune responses [111, 112].

Epitopes are classified as linear, consisting of contiguous amino acid sequences, or conformational, formed by non-contiguous residues in the antigen’s 3D structure [113]. Recent advancements have shifted from antibody-agnostic to antibody-aware approaches, incorporating structural and physicochemical features to improve prediction accuracy [112, 113]. Additionally, targeting functional or conserved epitopes enhances therapeutic efficacy, while epitope-specific antibodies improve diagnostic precision [112].

Accurate epitope identification relies on a combination of experimental and computational techniques, each offering distinct advantages in antibody and vaccine development [111, 114, 115]. Experimental methods, including X-ray crystallography, NMR spectroscopy, peptide ELISAs, and mass spectrometry, provide direct evidence of antibody binding [111]. Meanwhile, computational approaches such as ML, molecular docking, and bioinformatics tools offer scalable and cost-effective predictions [111]. Peptide scanning and alanine scanning mutagenesis further refine linear epitope identification by mapping critical binding sites with high precision [116, 117].

Computational epitope prediction tools fall into three main categories. Sequence-based methods, such as BepiPred [118] and ABCpred [119], focus on identifying linear epitopes by analyzing contiguous amino acid sequences. Structure-based approaches, including EpiPred [120] and EpiMap [117], predict conformational epitopes by considering the three-dimensional structure of the antigen. Additionally, hybrid models like DiscoTope [121] and ElliPro [122] integrate both sequence and structural data to enhance predictive accuracy, making them more effective in identifying epitopes with complex binding patterns.

Advanced ML-based tools, including DeepAb [88] and EpitopeVec [123], leverage large datasets to enhance predictive accuracy. Despite these advancements, epitope prediction remains challenging due to factors such as antigen conformational flexibility, limited training data, cross-reactivity, and epitope accessibility [111, 113]. Overcoming these limitations requires integrating experimental validation, refining computational algorithms, and expanding diverse training datasets to improve accuracy and reliability.

The Immune Epitope Database Analysis Resource (IEDB-AR) offers tools like TepiTool (for T cell epitope prediction), MHC-NP (for MHC ligand identification), and CD4EpiScore (for evaluating CD4 T cell reactivity) for B and T cell epitope prediction [124]. Several ML approaches significantly enhances epitope prediction, reducing the time and cost of traditional experimental methods [113]. These methods can predict both B-cell and T-cell epitopes, making them crucial for vaccine design and therapeutic antibody development [113]. Linear epitopes, consisting of contiguous amino acids, are predicted using sequence-based models like SVMs and RNNs, while conformational epitopes, formed by spatially close but non-contiguous residues, require models that integrate structural and sequence data to predict 3D configurations [113]. These methods streamline epitope-based peptide vaccine (EBPV) design by efficiently identifying immune-stimulating epitopes [113]. SVMs predict linear epitopes using sequence-based properties, while neural networks like RNNs, CNNs, and FNNs excel in both linear and conformational epitope prediction, with CNNs effectively capturing spatial relationships. Ensemble methods, such as random forests and gradient boosting, enhance accuracy for heterogeneous epitope datasets. Feature engineering further improves ML models by incorporating amino acid properties, hydrophobicity, charge, PCA, and evolutionary alignments to boost performance and precision [113]. ABCpred, an RNN-based model trained on 700 B-cell epitopes, predicts continuous B-cell epitopes with 65.93% accuracy, surpassing traditional methods and assisting vaccine and diagnostic research [119, 125, 126]. A biosupport vector machine (bSVM) achieved 90.31% accuracy in T-cell epitope prediction, outperforming traditional SVMs in identifying immune-relevant epitopes [127]. BCPred, an SVM-based model, improves linear B-cell epitope prediction with an AUC of 0.758, exceeding ABCpred and AAP in accuracy [128]. A logistic regression model using B-factor and RASA enhances discontinuous B-cell epitope prediction, outperforming DiscoTope and BEpro in sensitivity and AUC scores [129]. EPMLR, a multiple linear regression model, achieved 81.8% sensitivity and an AUC of 0.728 for linear B-cell epitope prediction [118, 128, 130]. Bagging meta decision trees (MDTs) integrated classifier outputs to improve conformational epitope predictions, reducing overfitting and surpassing 12 predictors, including SEPPA and DiscoTope [131]. A decision tree model also advanced metalloendopeptidase epitope prediction by identifying features like charged residues and physicochemical properties, validated experimentally to neutralize Atroxlysin-I's hemorrhagic activity [132]. Re-epitoping, a targeted antibody design approach, re-engineered antibodies like an IL-17A antibody for inflammatory diseases. Using ML to predict key binding contacts, it was validated with crystal structures, demonstrating its value in therapeutic antibody development [133]. Epitope3D, which uses graph-based signatures, predicts conformational B-cell epitopes with an MCC of 0.56 and F1 score of 0.57, supporting vaccine design and diagnostics [134].

Additionally, NetMHC, NetMHCpan, NetMHCII, and NetMHCIIpan are neural network–based computational tools that predict peptide binding to Major Histocompatibility Complex (MHC) molecules—an essential step in T-cell epitope identification and immunogenicity [124, 135–137]. NetMHC and NetMHCpan (including “pan” for a universal model) focus on MHC class I (CD8⁺ T‐cell) epitopes, while NetMHCII and NetMHCIIpan target MHC class II (CD4⁺ T‐cell) epitopes; each pair uses quantitative binding affinity data, with the “pan” versions able to predict across a broad range of MHC alleles [124, 135–137].

### Antibody structure predictions and design

Antibody structure prediction and design are integral to the rational development of therapeutic, diagnostic, and research antibodies, enabling the optimization of binding affinity, specificity, stability, and developability [10]. By predicting the 3D structure of antibodies and their antigen complexes, researchers can pinpoint critical residues in CDRs and engineer them to enhance interactions [138]. Structural insights also facilitate strategies to reduce immunogenicity, such as humanizing murine antibodies, and improve stability by addressing issues like aggregation and poor solubility [97, 138]. Additionally, computational tools enable de novo antibody design for novel or challenging targets, accelerating the discovery process by screening large libraries in silico [126–129]. This approach offers a cost-effective and efficient alternative to traditional methods like hybridoma technology or phage display.

ML and DL approaches complement experimental methods in antibody discovery, design, and optimization by reducing costs and addressing the limitations of traditional structure prediction. [37, 112]. Tools such as Rosetta [82, 143, 144], AlphaFold2 and 3 [28, 145, 146], DeepAb [88], ABlooper [147], and DeepH3 [148] have contributed to advances in protein and antibody structure prediction, supporting the use of structural data in antibody design and optimization. These computational tools leverage AI and ML to improve understanding of protein folding and interactions, aiding in the prediction of antibody structures with potential for high affinity. However, challenges remain in fully predicting and optimizing antibody properties, requiring further validation and refinement. Rosetta [82, 143, 144] facilitates macromolecular modeling, aiding in antibody-antigen complex prediction and affinity optimization. AlphaFold2 and AlphaFold3 revolutionized protein folding predictions, providing high-accuracy models that inform experimental designs [28, 145, 146]. DeepAb [88] specializes in antibody structure prediction, while ABlooper [147] focuses on CDRs, refining binding specificity. DeepH3 [148] improves modeling of CDR-H3 loops, which are highly variable yet crucial for specificity and affinity. These tools streamline antibody design by bridging sequence-to-structure predictions, enabling high-throughput screening, and guiding mutagenesis for affinity maturation, significantly enhancing therapeutic antibody development. Despite challenges like model interpretability, data completeness, and VH-VL pairing, these advancements facilitate the study design, development, and optimization of computationally designed antibodies [34, 149].

SVMs were initially used to predict protein structural classes based on amino acid composition, categorizing proteins into classes such as all-α, all-β, α/β, and α + β using SCOP database. By employing polynomial and Gaussian RBF kernels, SVMs achieved 100% accuracy in self-consistency tests and 74.5% in jackknife cross-validation, outperforming neural networks in both accuracy and generalization [150]. AbCPE, a multi-label classification algorithm, predicts antibody classes (IgG, IgE, IgA, IgM) binding to specific B-cell epitopes using methods like Binary Relevance and Label Powerset with Random Forest and AdaBoost classifiers. It demonstrated high accuracy, achieving a Hamming Loss of 0.1074 on test data and 0.036 for IgG-binding predictions, excelling in tasks such as SARS-CoV-2 epitope prediction. As the first multi-label approach in this domain, AbCPE marks a significant advancement in immune-informatics, aiding vaccine development, therapeutic antibody design, and diagnostics [151].

Computational methods for antibody structure prediction face challenges in modeling variable regions, particularly the CDRH3 region, due to its length, flexibility, and the impact of VH-VL chain orientation on binding. Tools like Rosetta address these challenges by using template-based approaches for canonical CDR loops and de novo methods for CDRH3 loop prediction, refining VH-VL orientation to improve docking accuracy [82, 143, 144]. RosettaAntibody, a high-resolution homology modeling protocol, achieves a median RMSD of approximately 1.5 Å for antigen-binding pockets in benchmark studies, though its accuracy varies based on antibody complexity. It faces challenges in accurately modeling long CDRH3 loops, a common limitation in homology-based approaches. While its docking accuracy is moderate, it contributes to antibody design by providing structural models that inform stability and binding affinity optimization in combination with other computational and experimental techniques [152]. AbPredict, another Rosetta-based tool, assembles variable domain fragments without relying on homology but faces difficulties with rare loop lengths. New AI models such as DeepAb, DeepH3, and ABlooper offer faster, template-free 3D structure predictions, facilitating high-throughput therapeutic antibody screening [153].

OptMAVEn-2.0 is an advanced computational tool for designing antibody variable regions targeting specific epitopes. It addresses inefficiencies of its predecessor by using k-means clustering, humanization measures, and Modular Antibody Parts (MAPs) to create high-affinity antibodies, validated through molecular dynamics simulations. Its scalability makes it applicable to diverse antigens, including those from infectious diseases [141]. Gradient Boosting Machine (GBM) models enhance structural cluster prediction for non-H3 CDRs, increasing accuracy from 79% to 88.16% by integrating sequence features and outperforming traditional methods. Despite challenges such as data sparsity and cluster imbalances, synthetic data and semi-unsupervised learning approaches may further enhance these models [154].

SCALOP is a sequence-based tool for predicting CDR canonical forms, achieving 89.47% accuracy and processing 100 sequences in 0.29 s, making it ideal for large-scale antibody repertoire analysis and immunological research [155]. Recent studies highlight the structural significance of the DE loop, traditionally considered a framework region. Variations in the DE loop, driven by germline and somatic mutations, stabilize CDRs and enhance antigen-binding affinity, playing a crucial role in improving binding specificity, particularly in broadly neutralizing antibodies against HIV [156].

AbAgIntPre, a CNN-based tool, predicts antibody-antigen interactions with an AUC of 0.82 on SARS-CoV datasets [157]. A reinforcement learning framework further optimizes CDRH3 sequences for antigen specificity using methods such as Fitness Buffer and Q-Ensemble Stability, outperforming methods like Structured Q-learning and Bayesian Optimization [87]. ABDPO (Antibody Direct Preference Optimization) addresses antibody design as a sequence-structure co-design problem, using pretrained diffusion models and residue-level energy metrics to optimize CDR configurations. It resolves energy conflicts with gradient surgery, surpassing DiffAb and MEAN in reducing structural clashes and accelerating therapeutic antibody development with high functionality and natural structures [158].

### Antigen–antibody interactions

Antigen–antibody interactions are essential for the immune response and serve as the foundation for therapeutic and diagnostic antibody design [159]. These interactions involve the binding of antibodies (immunoglobulins) to specific antigens, such as pathogens or toxins, with high specificity, where an antibody’s variable region recognizes a distinct epitope [38, 83, 159]. Binding occurs through non-covalent forces, including hydrogen bonds, ionic bonds, Van der Waals interactions, and hydrophobic interactions, ensuring strong yet reversible attachment [160]. The induced fit model explains how binding can induce conformational changes in both the antibody and antigen, enhancing specificity and affinity [159]. Additionally, these interactions are characterized by affinity (binding strength at a single site), avidity (multivalent binding effects), and structural complementarity, involving shape, hydrogen bonding, electrostatics, and hydrophobicity [161–163]. A deep understanding of these factors enables the rational engineering of antibodies with improved specificity, affinity, and stability, optimizing their application in immune defense, diagnostics, and therapy.

Researchers developed an SVM model to predict distance between antibody interface residues and antigens by using structural data from 37 antibody-antigen complexes in the Protein Data Bank (PDB). The model achieved up to 99% accuracy in predicting distance ranges (e.g., 8, 10, 12 Å) during validation, with improved performance for larger sequence patch sizes. Additionally, it classified antigen types (protein vs. non-protein) based on residue composition, aiding applications in epitope mapping, drug development, and vaccine design [164]. A method utilizing 3D Zernike Descriptors (3DZDs) and SVM classification accurately predicted antigen-binding regions (paratopes) on antibodies by incorporating geometric and physicochemical properties. It outperformed tools such as Paratome, Antibody i-Patch, and Parapred and was validated using Receiver Operating Characteristic (ROC) and Precision-Recall (PR) analyses. This approach optimized paratopes to enhanced antigen affinity and specificity while minimizing the need for extensive mutagenesis. It also demonstrated potential for antigen-specific predictions and integration with epitope and docking algorithms, enabling comprehensive interaction modeling [165].

A ML framework was developed for predicting antibody binding properties by incorporating data preprocessing, dimensionality reduction, and classification models. Using data from patent EP2275449B1, preprocessing included amino acid encoding and data balancing with Synthetic Minority Over-sampling Technique (SMOTE). Among six classifiers, Random Forest performed the best, achieving 97% accuracy for soluble BLyS and 83% for membrane-bound BLyS, excelling in precision, recall, and F-score [166]. Protein–protein interaction site prediction was improved using the XGBoost algorithm, which addressed imbalanced datasets with Repeated Edited Nearest Neighbor (RENN) and Instance Hardness Threshold (IHT) methods. By leveraging evolutionary conservation-based features, the model achieved 80.7% accuracy and a Matthews Correlation Coefficient (MCC) of 0.614 with IHT, outperforming traditional approaches. This method shows potential for large-scale protein interaction analysis, drug discovery, and cellular function studies [167].

A ML method predicted antibody-antigen binding directly from sequence data without requiring 3D structural information. Using Weighted k-NN and Random Forest models, it achieved 76% accuracy on a dataset of 600 computationally docked antibodies and 4441 interactions from the CoV-AbDab database. Features such as physicochemical properties and sequence metrics, refined using the BLOSUM62 matrix, improved prediction accuracy. While this approach advance immune repertoire analysis and antibody engineering, it underscores the need for more diverse, experimentally validated datasets [168]. The Antibody Random Forest Classifier (AbRFC) was developed to predict non-deleterious mutations in CDRs, improving antibody affinity. By leveraging structural and physicochemical features, AbRFC outperformed Graph Neural Networks (GNNs) and large language models (LLMs). Experimental validation demonstrated a 1,000-fold increase in SARS-CoV-2 antibody binding affinity against Omicron variants after minimal wet-lab screening [169]. A ML-guided platform incorporating AbRFC optimized antibody design by integrating computational prediction with experimental workflows. This platform enhanced antigen-binding affinity and developability using binary classification and advanced feature engineering, outperforming DL methods on small datasets. The iterative lab-in-a-loop framework achieved a two-order-of-magnitude improvement in binding affinity for anti-SARS-CoV-2 mAbs, demonstrating synergistic neutralization against Omicron variants and offering a scalable solution for therapeutic development against evolving pathogens [170].

## Advancing antibody therapeutics with AI

Recent advancements in AI have revolutionized antibody discovery, design, and development. Traditional methods, such as phage or yeast display and animal immunization, are limited by biological and chemical constraints [35, 36, 171]. In the late twentieth century, computational biology introduced techniques like high-throughput virtual screening (HTVS), molecular docking, and molecular dynamics simulations, which were initially constrained by computational power [25, 172]. Improvements in hardware and processing technologies have enabled the development of more efficient algorithms, fostering progress in ML, DL, and AI, which further advanced antibody design [173].

ML and DL, subsets of AI, are powerful tools for analyzing large datasets. ML includes supervised learning (predicting outcomes from labeled data), unsupervised learning (finding patterns in unlabeled data), and reinforcement learning (goal-oriented learning through rewards and penalties) [174, 175]. DL, a subset of ML, utilizes deep neural networks to handle complex tasks but is computationally demanding and less interpretable due to its hidden layers [176, 177]. Both ML and DL have significant potential in drug design, particularly in structure-based small-molecule and antibody design using techniques like CNNs and RNNs [176–179].

Generative models like GANs, VAEs, and reinforcement learning (RL) are transforming drug design by identifying molecular patterns and enabling multi-objective optimization for properties such as drug-likeness, bioactivity, and pharmacokinetics [36, 180, 181]. These models have demonstrated success in creating molecules with high binding affinity, multi-target activity, and optimal ADMET profiles [36, 180, 181]. Such examples include generative tensorial reinforcement learning (GENTRL) and Policy Gradient for Forward Synthesis (PGFS), which identified DDR1 kinase inhibitors optimized for binding affinity and approved for preclinical testing within 46 days [182, 183]. Additionally, deep generative models (DGMs) in poly-pharmacology designed compounds targeting GSK3β and JNK3, enhancing both efficacy and safety [184]. Platforms such as Chemistry42 have advanced AI-driven drug design, exemplified by INS018_055 for idiopathic pulmonary fibrosis, which has progressed to Phase II trials [185, 186]. RNN-based models have also contributed, such as the generation of RIPK1 inhibitor RI-962, which exhibits potent in vitro and in vivo activity against inflammatory diseases [187]. Fragment-based design algorithms like RationaleRL optimize bioactivity for multiple targets, while flow-based models such as MoFlow efficiently map molecular graphs [184, 188]. PaccMann integrates 3D structural data for precise ligand–protein interactions, enhancing molecular generation accuracy and minimize reconstruction errors [36, 180, 181]. These advancements highlight AI's potential to streamline drug discovery, design, and optimization.

Transformer-based models, leveraging attention mechanisms, have significantly advanced sequential data processing for SMILES prediction, multitask learning, and chemical and antibody structure design and optimization [189–191]. Tools like AlphaPanda combine transformers models, 3D CNNs, and diffusion models to generate antibody structures, effectively capturing global sequence and local structural information for improved design [192]. In a case study, transformer and GAN models were used to diversify CDR3 regions, achieving an 87% success rate in identifying high-affinity antibodies [193]. AB-Gen, a generative pre-trained transformer with deep reinforcement learning, was employed to design HER2-targeting antibody libraries, with critical residues validated through simulations [194]. AntiBERTa, a transformer-based language model trained on 57 million human BCR sequences, excels in paratope prediction and outperforms tools such as Parapred [195], ProABC-2 [196], ProtBERT [197] and Sapiens [95]. It supports structure prediction, humanization, and BCR analysis, advancing antibody discovery, diagnostics, and engineering through self-supervised learning [198]. LLMs like ESM-1v predict the impact of mutations on protein function with accuracy comparable to supervised models using pre-trained data [199]. mCSM-AB2, a web server for antibody engineering, predicts mutation effects on antibody-antigen binding affinity using graph-based signatures, evolutionary data and energy-based features. It achieves high accuracy, with Pearson's correlation coefficients of 0.73 in training and 0.77 in blind tests, surpassing FoldX [101]. Based on a dataset of 1,810 mutations from AB-BIND [200], PROXiMATE [201], and SKEMPI2.0 [202], mCSM-AB2 aids in affinity maturation and large-scale mutation analysis, supporting therapeutic antibody development [203]. These advancements highlight the transformative role of transformer models and AI-driven tools in antibody engineering and drug design.

A ML framework predicts scFv poly-reactivity by combining antibody properties with NLP-based protein descriptors, identifying factors such as CDR2 loop net charge, specific residues, and loop length. Using models like GBM and Random Forest, it achieves high accuracy (AUC 0.840) and integrates aggregation scores and SASA via trRosetta for efficient scFv screening, advancing antibody and enzyme design [204]. Another framework leverages pretrained language models and Bayesian optimization to enhance scFv binding affinities, achieving up to 99% success, including a 28.7-fold improvement for heavy-chain scFvs. With up to 23 mutations, these diverse libraries outperform traditional methods like position-specific scoring matrix (PSSM), enabling advanced antibody engineering and multi-objective optimization [205].

NLP models such as ProtT5 [206], Transformer-XL [207], and BERT [208] are increasingly adapted in bioinformatics to analyze protein sequences without relying on evolutionary data [206]. Trained on large protein databases, these models accurately capture biophysical features like secondary structure and localization [206]. ProtT5, for instance, achieved state-of-the-art accuracy (81–87%) in secondary structure prediction while avoiding costly evolutionary data, enabling large-scale analyses like processing the entire human proteome in under an hour. These advancements demonstrate the potential of pre-trained language models in protein engineering, drug discovery, and functional annotation [206].

Structured Q-learning (SQL), an advanced reinforcement learning method, optimizes combinatorial structures in antibody design. Using Variable Allocation Markov Decision Process (VAMP) and structural exploration operators, SQL efficiently optimizes CDRH3 sequences for antigen binding, outperforming methods like policy gradients and simulated annealing. It generated over 300 unique optimal CDRH3 sequences per target with superior binding energy scores, including for SARS-CoV spike proteins, enhancing antibody diversity and quality while reducing computational demands [209]. AbBERT, trained on over 50 million antibody sequences from the Observed Antibody Space (OAS) dataset, integrates language models with sequence-structure co-design, achieving state-of-the-art accuracy in amino acid recovery (40.35%) and structural prediction (RMSD 1.62) for CDR-H3, enabling antigen-specific antibody design [210].

ReprogBERT repurposes a pretrained English BERT model for protein sequence infilling, specifically targeting antibody CDR design. It generates highly diverse CDR sequences, achieving a two-fold increase in diversity over baseline models while maintaining structural integrity and sequence naturalness. The model demonstrates superior performance, with lower perplexity scores and higher diversity in generated sequences. Structural validation using AlphaFold confirms the biological relevance of their outputs. These results suggest ReprogBERT’s potential for on-demand antibody design, making it a valuable tool for therapeutic and diagnostic applications [211]. AbImmPred predicts therapeutic antibody immunogenicity using AntiBERTy, a pre-trained antibody language model that extracts sequence features without 3D structural data [212]. Using AutoGluon, it achieves high accuracy (0.7273) with improved recall (0.9375), precision (0.7500), and F1-score (0.8333) over methods like PITHA [213]. This tool offers a cost-effective solution for early-stage antibody screening in computational immunology and therapeutic development [212].

Other LLMs and GPT inspired models such as ProtGPT2 [214], AbGPT [139], IgLM [215], AB-Gen [194], AntiBARTy Diffusion [216], and pAbT5 [217] have further advanced antibody design and engineering (discussed in later sections). These tools streamline therapeutic development by integrating AI-driven sequence and structure optimization, addressing immunological challenges, and enhancing protein engineering.

AI-driven methods have revolutionized protein structure prediction and interaction analysis, significantly advancing protein-based therapeutic development [218]. These algorithms excel at predicting structures, binding sites, and generating novel antibody sequences, achieving binding rates over 10% [34, 142]. By leveraging large datasets, ML, and NLP, AI enhances immunology, diagnostics, and drug discovery, accelerating the development of antibody-based therapeutics [219]. Table 1 summarizes the contributions of ML, DL, and generative AI to antibody design and optimization.

**Table 1:** Overview of Major AI Algorithms with Description and Applications at a Glance

Major AI Algorithms and subtypes	Description	Applications in Antibody Designing
**Supervised Learning (SL)**	SL involves training a model on labeled data	
**Linear Regression**	Used for predicting continuous values	B-cell epitope prediction [130]
**Logistic Regression**	Used for binary classification problems	Discontinuous B-Cell epitopes prediction [129]
**Support Vector Machines (SVM)**	Works well for classification and regression tasks	B-Cell and T-Cell epitopes prediction [113, 127], antibody interface (and antigen interaction) prediction [164, 165], prediction of protein structural classes [150], protein design [100, 149]
**k-Nearest Neighbors (k-NN)**	A non-parametric technique for classification and regression	Antibody aggregation and viscosity prediction [110], antibody-antigen binding [168]
**Naive Bayes**	Based on Bayes’ theorem, used for classification tasks	Antibody optimization [205] design and development of antibodies and immunogens [153], antibody properties predictions [166]
**Decision Trees**	Used for classification and regression by dividing data into subsets based on feature values	Viscosity behavior [109], epitope prediction for B-Cell antibodies [131]
**Unsupervised Learning**	Unsupervised learning involves training a model on data that lacks labeled outcomes	
**k-Means Clustering**	Partitions data into k distinct clusters based on feature similarity	De-novo design of variable antibody [141]
**Hierarchical Clustering**	Builds a hierarchy of clusters either by a bottom-up or top-down approach	Analysis of variation in relative VL-VH orientations [152]
**DBSCAN (Density-Based Spatial Clustering of Applications with Noise)**	Clusters data based on the density of data points	Clustering loop structures [156]
**Principal Component Analysis (PCA)**	Reduces dimensionality of data while retaining most of the variance	Dimensionality reduction [34, 205]
**t-Distributed Stochastic Neighbor Embedding (t-SNE)**	Non-linear dimensionality reduction technique for visualizing high-dimensional data	High-dimensional scFv sequences visualization [205]
**Semi-Supervised Learning**	Semi-supervised learning algorithms leverage a combination of labeled and unlabeled data for training	
**Self-Training**	Iteratively labels the unlabeled data using predictions from the model	Training a large antibody-specific language model [198, 206, 210, 211]
**Reinforcement Learning**	Reinforcement learning trains models to make decisions by rewarding desired actions	
**Q-Learning**	A model-free algorithm for estimating the value of an action within a specific state	Optimize amino acid sequence binding [87, 209]
**Deep Q-Networks (DQN)**	Combines Q-learning with deep neural networks	Optimize amino acid sequence binding [87]
**Policy Gradient Methods**	Directly optimize the policy by gradient ascent	Fine tuning in alignment of generative antibody models [158]
**Proximal Policy Optimization (PPO)**	A policy gradient method that ensures updates are not too large, improving stability	Computational thinking and optimal solution [220]
**Ensemble Learning**	Ensemble methods merge multiple models to boost performance	
**Bagging (Bootstrap Aggregating)**	Reduces variance by averaging the predictions of multiple models (e.g., Random Forests)	B-cell epitope identification [132], antibody engineering and small data problem [170]
**Gradient Boosting Machines (GBM)**	Combines weak learners (typically decision trees) to form a strong predictive model	Protein solubility prediction [102], learn the structural clusters of non-H3 CDRs from sequence alone and feature selection, group and classify non-H3 CDRs into structural clusters [154, 155, 204]
**AdaBoost**	AdaBoost is an ensemble algorithm that strengthens weak classifiers by emphasizing misclassified instances	Enhancing prediction efficiency in epitope prediction [151] conformational epitopes prediction [134]
**XGBoost**	XGBoost is a powerful, scalable ML algorithm that uses gradient boosting to improve the performance of decision trees by optimizing for speed and efficiency	Effects of mutations on antibody binding affinity [203], protein–protein interaction sites prediction [167], immunogenicity prediction [212], antibody developability properties prediction [90]
**LightGBM**	LightGBM is a fast, efficient gradient boosting framework optimized for large datasets and high-speed tree-based learning	Predicting protein–protein interactions [221], immunogenicity prediction [212]
**CatBoost**	CatBoost is a gradient boosting library designed to handle categorical features efficiently, providing high accuracy and fast performance without extensive data preprocessing	Immunogenicity prediction [212]
**Random Forests**	An ensemble technique that boosts accuracy with multiple decision trees	Design of epitope-specific functional antibodies [133] antibody affinity enhancement [169]
**Deep Learning/ Generative Algorithms**	DL uses multi-layered neural networks	
**Convolutional Neural Networks (CNN)**	Used primarily for image and video recognition	Predict the bioactivity of small molecules [222] generation of synthetic antibodies and binders [223], screening of therapeutic antibody viscosity [224], amino acid sequence based antibody–antigen interactions identification [157]
**Recurrent Neural Networks (RNN)**	Ideal for sequential data like time series and natural language	Sequence representation [88], peptide HLA class binding prediction [225], B cell epitope prediction [119]
**Long Short-Term Memory (LSTM)**	A type of RNN that can capture long-term dependencies	Antibody design [226], binding sites prediction [227], optimization of antibody [98], protein–protein interaction sequence design [228, 229]
**Gated Recurrent Unit (GRU)**	It is a simplified RNN that captures temporal dependencies in sequential data using gating mechanisms	Antibody specificity prediction [31]
**Generative Adversarial Networks (GANs)**	GANs consist of two neural networks—a generator and a discriminator—trained together through adversarial interaction	Antibody optimization improvement [85], prediction and generation of synthetic antibody [223], humanoid antibody design [230, 231]
**Variational Autoencoders (VAE)**	VAEs are generative models that merge DL with Bayesian inference, featuring an encoder that maps data to a latent space and a decoder that reconstructs data from that latent space	Sequence patterns that are predictive of antigenic exposure [232], to learn the rules of VDJ recombination [233], 3D coordinate generation [234], assessment of antibody and nanobody nativeness [235], protein variants generation [236]
**Transformer**	The Transformer is a DL architecture that leverages self-attention to process input sequences in parallel, efficiently managing long-range dependencies and delivering top performance in NLP tasks	Novel antibody generation [193], antibody-specific language model [198], B-cell conformational epitope prediction [237], antibody library design [194], co-design of structures and sequences of antibodies [192], effect of mutation and secondary structure prediction [238], structure prediction and missing residues filling [239], generation of improved sequences followed by developability and reduced immunogenic risks [215], antibody sequence generation [240]
**BERT (Bidirectional Encoder Representations from Transformers)**	BERT is a pre-trained DL model that uses bidirectional self-attention to grasp word context within a sentence, excelling in NLP tasks by analyzing both left and right context simultaneously	Key binding residues identification and affinity maturation [241], per-residue or per-sequence protein predictions tasks [206], amino acid prediction in a given sequence at a specific position [242], for antibody sequence infilling [211], antibody language model [243], protein language model [206]
**GPT (Generative Pre-trained Transformer)**	GPT is a language model built on the Transformer architecture that generates human-like text. Pre-trained on extensive text data and fine-tuned for specific tasks, it excels in natural language understanding and generation by predicting the next word in a sequence	Antibody library design [194], to perform protein docking using flexible and site-specific options [244], de-novo protein sequences generation [214], protein language model [240]
**T5 (Text-to-Text Transfer Transformer)**	T5 is a flexible language model that frames all NLP tasks as text-to-text problems, using the Transformer architecture to convert input text into output text, enabling it to tackle tasks like translation, summarization, and question answering with a single approach	Antibody language model [243], protein language model [206]
**Hugging Face Transformers**	Provides pre-trained models for a variety of NLP tasks	Amino acid prediction in a given sequence at a specific position [242], large scale paired antibody language models [243], antibody generation [240], antibody design using generative language model [245], for to completing antibody sequences using language model [246]
**Conditional GAN (cGAN)**	cGAN is a type of Generative Adversarial Network that generates data conditioned on input data, allowing the generation of specific types of outputs, such as images or text, based on given labels or attributes	De-novo inspired protein design [247]
**Gaussian Processes (GPs)**	Gaussian Processes (GPs) are a non-parametric, Bayesian approach to modeling functions, providing predictions with a measure of uncertainty by defining a distribution over functions and using observed data to update this distribution for making predictions	To optimize the given antibody [205]
**Graph Neural Networks (GNN)**	GNNs are DL models that operate on graph-structured data, capturing node relationships and dependencies through iterative message passing and aggregation	Protein design [248], antibody binding and developability properties [248], ligand binding site prediction [249], antibody sequence-structure co-design [250], CDR loop structure prediction [147]
**Graph Convolutional Networks (GCN)**	GCNs are a type of GNN that extend convolutional operations to graphs, allowing them to aggregate information from neighboring nodes and learn representations that reflect both local and global graph structure	Predicting unseen antibodies’ neutralizability [251], paratope prediction [252]
**Graph Attention Networks (GAT)**	Graph Attention Networks (GATs) are a type of Graph Neural Network (GNN) that utilize attention mechanisms to weigh the importance of neighboring nodes. This allows the model to focus on the most relevant parts of the graph when learning node representations	Antibody binding and developability properties prediction [248]
**Autoregressive Models**	These models generate data one step at a time, with each step conditioned on the previous ones	Protein design and variant prediction [253], antibody sequence design [254], Few-Shot learners using language models [255], learned potential based protein sequence design [256], antibody design using generative language modeling [245]
**Flow-based Models**	Flow-based models learn the data distribution by gradually converting a simple distribution, like a Gaussian, into the data's distribution through a sequence of reversible transformations	CDR-designing [257]

## AI based early drug discoveries and platforms

AI-driven drug discovery has demonstrated significant success, beginning with BenevolentAI, which repurposed baricitinib for COVID-19, significantly reducing mortality rates in hospitalized patients [258, 259]. Following this, collaborations between Sumitomo Dainippon Pharma and Exscientia led to the rapid development of candidates such as DSP-1181 (targeting OCD) and DSP-2342 (for psychiatric diseases) within just 12 months, with Exscientia’s AI-driven platform accelerating synthesis and testing cycles [260, 261]. In 2021, Exscientia introduced an AI-designed A2A receptor antagonist, which entered clinical trials for advanced solid tumors [274]. Another key development was EXS-21546, co-developed with Evotec, targeting cancer by inhibiting the A2A receptor to enhance immune responses. Currently in Phase I/II trials (NCT05920408), it shows promise for treating solid tumors. Exscientia further introduced the adenosine burden score (ABS), an immuno-oncology biomarker predicting patient responses to EXS-21546, particularly in combination with checkpoint inhibitors [262].

AI has also accelerated drug discovery in neurological and inflammatory diseases, with BenevolentAI advancing candidates such as BEN-8744 (Phase I, NCT06118385) for ulcerative colitis, BEN-28010 for glioblastoma multiforme, and BEN-34712 for amyotrophic lateral sclerosis (ALS), which demonstrated significant preclinical efficacy [258, 259]. Beyond small-molecule drug discovery, AI is transforming antibody development, offering efficiency, accuracy, and cost reduction in therapeutic design [38]. AI-driven data analysis and pattern recognition enable precise identification of complex antibody-antigen interactions, enhancing therapeutic antibody design [38, 211, 263–269].

**Fig. 5:**
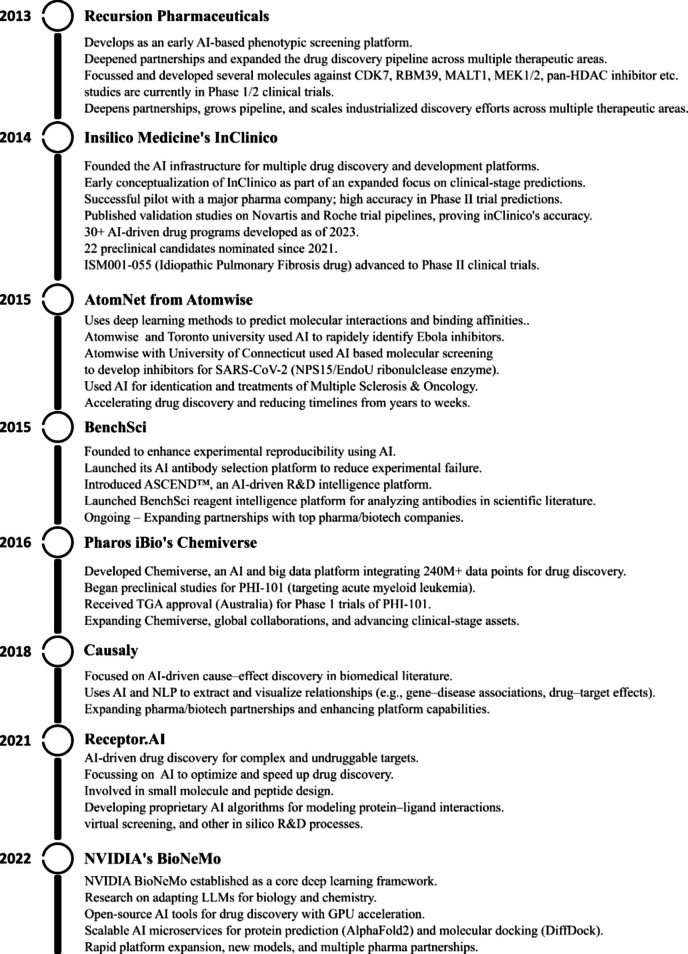
Selected AI platforms and their importance in AI based drug discovery and design

Several AI-powered platforms play crucial roles in antibody selection, modeling, and optimization. BenchSci (https://www.benchsci.com/) accelerates preclinical research, Atomwise's AtomNet (https://www.atomwise.com/how-we-do-it/) applies DL to molecular discovery, and DeepMind's AlphaFold (https://deepmind.google/) revolutionizes protein structure prediction [270]. Other platforms, such as Causaly (https://www.causaly.com/), Pharos iBio's Chemiverse (https://www.pharosibio.com/en/), Insilico Medicine's InClinico (https://insilico.com/), and Recursion Pharmaceuticals (https://www.recursion.com/), automate protein–ligand interaction analysis, clinical trial outcome predictions, and drug development acceleration. The Binding Site-Augmented DTA model, developed at the University of Central Florida, demonstrates how DL refines drug-target affinity predictions [269]. Generative biology, integrating AI with lab science, is transforming drug development by designing therapies that surpass natural proteins. Companies like Amgen and BigHat Biosciences use AI-driven wet lab experiments to enhance drug effectiveness, while AI-powered platforms such as NVIDIA's BioNeMo (https://www.nvidia.com/en-us/clara/bionemo/), BenchSci's ASCEND (https://knowledge.benchsci.com/home/platform-overview), and Receptor.AI (https://www.receptor.ai/) streamline drug optimization, reducing time and costs. Figure 5 showed the importance of several selected AI based platforms in drug discovery and design.

AI-driven antibody drug design has led to multiple candidates progressing into clinical trials. Biolojic Design’s AU-007, targeting IL-2 to enhance cancer immune responses, is currently undergoing trials [271]. AbCellera Biologics developed bamlanivimab for COVID-19, which entered Phase II trials. Adagene’s AI-derived antibodies, including ADG-106, 104, 116, 126, BC-006, and Adimab’s PM-1022, are being developed for oncology. Compugen's bapotulimab, COM-701, and COM-902, along with HiFiBiO Therapeutics' antibodies (HFB-301001, HFB-200301, and HFB-30132A for COVID-19), are undergoing Phase I or II trials, demonstrating AI’s transformative role in antibody drug design [272]. NL-201 by Neoleukin Therapeutics, a de novo protein mimicking IL-2 and IL-15, reached Phase 1 trials for cancer before discontinuation in 2022 [273].

Several industrial collaborations have accelerated AI-driven drug discovery. Recursion Pharmaceuticals and Exscientia partnered to scale AI-driven research, while Absci and AstraZeneca are co-developing an AI-designed oncology antibody. Antiverse and Nxera Pharma (formerly Sosei Heptares) are creating GPCR-targeted antibodies, integrating Antiverse's generative AI with NxWave™, a GPCR validation platform [274, 275]. AION Labs launched DenovAI, a startup focused on de novo antibody design, leveraging AI and biophysics to develop high-affinity antibodies and miniprotein binders, supported by Pfizer, AstraZeneca, Merck, Teva, AWS, and the Israel Biotech Fund [276]. These partnerships demonstrate AI’s growing role in drug discovery, accelerating therapeutic development and improving clinical success rates. As AI continues to evolve, its integration with experimental research is expected to drive unprecedented advancements in precision medicine and personalized therapeutics. AI-assisted drug candidates in clinical investigations are extensively reviewed elsewhere [36].

## AIs application in antibody design and optimization

mAbs are essential for immune responses due to their broad functionality and prolonged half-lives [277]. However, stability issues vary across formats; full-sized antibodies often lack thermodynamic stability despite being kinetically stable, whereas single-chain variable fragments (scFvs) typically exhibit poor to moderate stability [278]. Advances in antibody engineering, such as humanization, point mutations, and stable framework grafting, have improved stability, folding efficiency, and reduced immunogenicity [277–279]. Modern antibody design integrates computational and experimental techniques for precision and efficiency. Computational tools like protein modeling, molecular docking, and dynamics predict structures, optimize electrostatics, and identify high-affinity mutations, particularly in regions like CDR-H3 [280, 281]. Structure-guided approaches analyze and optimizes antibody-antigen interactions using hydrogen bonding, electrostatics, and shape complementarity, while experimental methods like phage display, mutagenesis, and X-ray crystallography validate these predictions [282, 283]. This synergy enhances binding affinity, reduces aggregation, and accelerates therapeutic development, as exemplified by affinity improvements in cetuximab (140-fold affinity improvement) and engineered anti-VEGF antibodies [280, 281, 283]. These methods are essential in modern antibody engineering, focusing on principles like stabilizing framework-CDR interactions and preserving critical amino acids in the Fv backbone for stability and specificity [284]. Such designs achieve high binding specificity and stability, even with significant divergence from natural germlines, showcasing the effectiveness of computational approaches [284]. Computational approaches have also been used to predict beneficial mutations and redesign antibodies by altering a single CDR [80, 285, 286]. For instance, stepwise randomization of residues in CDR-H3 and CDR-L3, guided by computational docking, improved the binding affinities up to 25.7-fold, as seen with P96 mutations in CDR-L3. Additionally, efforts have focused on predicting antibody structures directly from amino acid sequences [28, 82]. These studies highlight the potential of computational methods to enhance antibody-binding properties. The primary goal of AI is to predict and automate the generation of CDR subsequences with properties essential for antigen binding and antibody effectiveness [42, 149]. Designing CDRs that target specific antigens is crucial for therapeutic antibody development but challenging due to the vast combinatorial space of over 20^60^ possible sequences [287]. Testing all CDR combinations experimentally is impractical, necessitating computational approaches. Traditional methods relying on biophysical energy functions are time-consuming and prone to local optima [288]. AI has transformed CDR design by enabling precise modeling and prediction, significantly accelerating the process. Beyond automated CDR prediction, AI also improves antibody stability, folding efficiency, and epitope-paratope interactions, particularly in accurately predicting CDR-H3 conformations.

Table 2 summarizes tools and models developed using AI, ML, generative AI, and LLM-based algorithms.

**Table 2:** Developed Tools and their Applications

S. No	Tools/Model	Year	Function/Role	GitHub link	Reference
Structural Prediction of CDRs					
**1**	AbFlex	2024	CDR design method with a given antibody-antigen complex	https://github.com/wsjeon92/AbFlex	[289]
**2**	AbDiffuser	2023	Generation of antibody 3D structures and sequences	Not Mentioned	[290]
**3**	ImmuneBuilder/ ABodyBuilder2/ NanoBodyBuilder2 TCRBuilder2	2023	Fv modelling, antibody structure modeling, TCR structure modelling, nanobody structure modelling	https://github.com/oxpig/ImmuneBuilder https://opig.stats.ox.ac.uk/webapps/sabdab-sabpred/sabpred	[291]
**4**	IgFold	2022	Fast, accurate antibody structure prediction	https://github.com/Graylab/IgFold	[24]
**5**	ABLooper	2022	Antibody CDR loop structure prediction	https://github.com/oxpig/ABlooper	[147]
**6**	DeepSCAb	2022	Prediction of antibody backbone and side-chain conformations	https://github.com/Graylab/DeepSCAb	[292]
**7**	DeepAb	2022	Antibody structure prediction	https://github.com/RosettaCommons/DeepAb	[88]
**8**	SMCDiff	2022	Protein backbones and motif-scaffolding	https://github.com/blt2114/ProtDiff_SMCDiff	[293]
**9**	DeepH3	2020	Prediction of CDR H3 loop	https://github.com/Graylab/deepH3-distances-orientations	[148]
**10**	RosettaAntibody	2018	Antibody modelling	https://www.rosettacommons.org/ https://github.com/RosettaCommons/rosetta	[82]
**11**	AlphaFold		Structure prediction and modelling	https://github.com/google-deepmind/alphafold	[28]
Optimization and Affinity improvement					
**12**	IgDiff	2024	De novo antibody design	Not Mentioned	[294]
**13**	AbGAN-LMG	2023	Higher-quality antibody libraries generation and optimization	https://github.com/Zhaowenbin98/AbGAN-LMG	[85]
**14**	Ens-Grad	2020	CDR design	https://github.com/gifford-lab/antibody-2019	[295]
**15**	OptMAVEn2.0	2018	De novo Design of Antibody Variable Region	https://github.com/maranasgroup/OptMAVEn_2.0	[141]
**16**	OptCDR	2010	CDR designing	http://maranas.che.psu.edu (link not working)	[80]
**17**	MEAN	2023	antibody design	https://github.com/THUNLP-MT/MEAN	
Generating CDR Libraries					
**18**	IgLM	2023	Generates full-length antibody sequences and Infilled CDR H3 loop libraries generated	https://github.com/Graylab/IgLM	[215]
**19**	reportBERT				
**Optimizing CDR Immunogenicity**					
**20**	EquiPocket	2023	Binding site prediction	Not Mentioned	[249]
**21**	AbAgIntPre	2022	Predict antibody-antigen interactions	http://www.zzdlab.com/AbAgIntPre/	[157]
**22**	DLAB	2022	Predict antibody–antigen binding for antigens/ binder/ Virtual screening/ non-binder classifier	https://github.com/oxpig/dlab-public	[296]
**23**	PECAN	2020	Predict binding interfaces on both antibodies and antigens	https://github.com/vamships/PECAN	[297]
Epitope and paratope prediction					
**24**	Paragraph	2023	Antibody paratope prediction	https://github.com/oxpig/Paragraph	[298]
**25**	SEMA	2022	B-cell conformational epitope prediction	https://github.com/AIRI-Institute/SEMAi	[237]
**26**	EPMP	2021	Joint epitope-paratope prediction	NA	[299]
Miscellanies					
**27**	AbSciBio	2024	De novo antibody design	https://github.com/AbSciBio/unlocking-de-novo-antibody-design	[142]
**28**	ProGen2	2023	Modeling evolutionary sequence distributions, creating novel sequences, and predicting protein fitness	https://github.com/salesforce/progen	[240]
**29**	Protpardelle	2023	Generative model for protein design	https://github.com/ProteinDesignLab/protpardelle	[300]
**30**	AbLang	2022	Restores the missing residues of antibody sequences	https://github.com/oxpig/AbLang	[246]
**31**	AbBERT-HMPN	2022	Generation of sequences and structures, focusing on the design of antigen-binding CDR-H3 regions	Not Mentioned	[210]
**32**	AntiBERTa	2022	Tracing B cell origins, quantifying immunogenicity, and predicting antibody binding sites	https://github.com/alchemab/antiberta	[198]
**33**	HERN (Hierarchical Equivariant Refinement Net- work)	2022	Antibody docking and design	https://github.com/wengong-jin/abdockgen	[301]
**34**	RoseTTAFold Diffusion (RFdiffusion)	2022	Enables the creation of complex, functional proteins from basic molecular conditions	https://github.com/RosettaCommons/RFdiffusion	[302]
**35**	AntiBERTy	2021	Understanding of immune repertoires/and affinity maturation/ insights into antigen binding	https://github.com/jeffreyruffolo/AntiBERTy	[241]
**36**	RefineGNN	2021	Optimization guided by specific properties to design new antibodies with enhanced neutralization capabilities	https://github.com/wengong-jin/RefineGNN	[250]
**37**	LSTM based study	2021 2022	Antibody design and affinity maturation Antibody Binding site prediction	Not Mentioned https://github.com/mpcrlab/AntibodyBindingPrediction	[226] [227]
**38**	Fold2Seq	2021	Designing protein sequences tailored to a specific target fold	https://github.com/IBM/fold2seq	[303]
**39**	UniRep	2019	Protein engineering and informatics	https://github.com/churchlab/UniRep	[304]

### AI in B cell epitope prediction, paratope identification, and antibody-antigen interactions

Epitopes are specific regions on antigens that antibodies recognize and bind to, playing a key role in specificity, binding affinity, and immune targeting of cancer cells [305]. They contribute to immune system targeting of cancer cells and may support long-term immunity through immune recognition. Designing antibodies to target specific epitopes involves challenges such as ensuring accessibility and overcoming tumor variability, making epitopes central to the precision and effectiveness of therapeutic antibodies in cancer treatment [107].

A 2007 benchmark study revealed that no tools for antibody-protein interactions achieved precision over 40% or recall beyond 46% [306]. Since then, a knowledge-based method using consensus structural data for CDRs and B-cell epitopes has been developed and refined [267, 305]. The need for better in silico B-cell epitope prediction tools and the value of combining computational and experimental methods were also emphasized [114]. Recent advances in predicting conformational B-cell epitopes offer potential for vaccines and therapies, but further improvements in accuracy could enhance immunotherapeutic drug development [305].

A model named SEMA was developed using a transfer learning approach with pretrained DL models, ESM-1v and ESM-IF1. The SEMA model, fine-tuned to predict antibody-antigen interactions, achieved a ROC AUC of 0.76 on an independent test set, demonstrating competitive performance compared to peer-reviewed tools [237]. Additionally, a DL-based framework using graph convolutions (to capture spatial proximity) and attention layers (to capture antibody-antigen context, utilizing transfer learning from general protein interactions) accurately predicts binding interfaces on antibodies and antigens, enhancing accuracy and providing interpretable insights into antibody-antigen interactions [297]. The structure-based paratope prediction tool, Paragraph, has demonstrated strong performance in paratope prediction, offering an alternative to existing state-of-the-art methods [298]. It was shown that epitope residues are distant and antigen-dependent, leading to the development of Para-EPMP and Epi-EPMP models. Para-EPMP processes antibody features sequentially using a graph structure, while Epi-EPMP incorporates structural features and GNN layers with contextual information from the related antibody [299]. Unlike the complex EPMP approach, Paragraph focuses solely on structural features for accurate paratope prediction. A structure-based framework known as Deep Learning for Antibodies (DLAB) was created to enable virtual screening of antibodies against antigens, even in the absence of known binders. DLAB improves antibody-antigen docking by refining pose ranking and demonstrating strong performance in identifying compatible pairings in validation studies. Case studies highlight DLAB’s effectiveness in detecting binding antibodies, indicating its potential to support and streamline aspects of the antibody drug discovery process [296]. Similarly, AbAgIntPre, a DL-driven approach, was designed to swiftly predict antibody-antigen interactions using amino acid sequences. Leveraging a Siamese-like CNN, it achieved an AUC of 0.82 on an independent test set, demonstrating strong predictive accuracy in a SARS-CoV dataset. These models are expected to complement traditional methods and provide computational insights that may improve aspects of antibody design [157]. These studies emphasize the critical role of accurate B-cell epitope prediction, paratope identification, and antibody-antigen interaction analysis in antibody engineering.

### AI applications in CDR prediction, generation and modeling

#### Structural Prediction of CDRs

DeepH3, a DL approach, predicts CDR H3 loop structures using inter-residue distances, orientations, and geometric potentials. It demonstrates improved structure identification accuracy by 32.1% and achieves a mean RMSD of 2.2 ± 1.1 Å in de novo predictions, outperforming traditional methods [148].

IgFold is a fast DL method for antibody structure prediction; it uses a pre-trained model on 558 million antibody sequences combined with graph networks to estimate backbone atom coordinates [24]. Its predictions are comparable to or, in some cases, surpass those of methods like AlphaFold, with significantly faster processing times, enabling large-scale studies. For instance, IgFold has been applied to predict structures for 1.4 million paired antibody sequences, vastly expanding insights into antibody diversity beyond experimentally resolved structures [24].

ImmuneBuilder is a toolkit for modeling immune-related proteins, featuring ABodyBuilder2 for antibodies, NanoBodyBuilder2 for nanobodies, and TCRBuilder2 for T-cell receptors [291]. ABodyBuilder2 predicts CDR-H3 loops with an RMSD of 2.81Å, slightly better than AlphaFold-Multimer, while NanoBodyBuilder2 achieves an RMSD of 2.89Å, surpassing AlphaFold2 by 0.55Å. ImmuneBuilder provides structure ensembles and error estimates, offering additional insights into prediction confidence [291].

ABlooper, an end-to-end DL tool, predicts CDR loop structures with a focus on the variable CDR-H3 loop. It provides rapid, reliable predictions with confidence estimates, achieving an average RMSD of 2.49 Å for CDR-H3, improving to 2.05 Å for the top 75% of high-confidence predictions on Rosetta Antibody Benchmark models. This tool contributes to advancements in antibody modeling, particularly for the challenging CDR-H3 loop [147].

AbFlex, an advanced antibody design model, addresses limitations in structural prediction and amino acid recovery. Over 38% of designed antibodies exhibited binding energies that were lower (indicative of stronger binding) compared to the wild-type in computational evaluations. Utilizing a data-efficient equivariant graph neural network and flexible CDR definitions with novel data augmentation, AbFlex enhances CDR predictions and antibody binding efficiency, advancing therapeutic antibody engineering [289].

AbDiffuser, an equivariant physics-informed diffusion model, was developed to improve antibody 3D structure and sequence generation. It features a novel protein structure representation, an innovative architecture for aligned proteins, and strong diffusion priors, enhancing denoising, handling sequence-length changes, and reducing memory complexity. Numerical experiments validated AbDiffuser’s ability to generate antibodies matching reference sequences and structures. Laboratory tests demonstrated expression for all 16 discovered HER2 antibodies, with 57.1% exhibiting tight binding characteristics [290].

DeepSCAb, a DL framework, predicts inter-residue geometries and side-chain dihedrals of antibody variable fragments using only sequence data. It excels with unknown backbone conformations, leveraging self-attention to detect conserved positions across species. DeepSCAb demonstrates competitive performance in identifying near-native structures and achieves accuracy comparable to rotamer repacking for side-chain prediction. This advancement enhances antibody structure prediction, aiding in antibody-antigen docking and therapeutic antibody design [292].

Graph-based and sequence-based models have been developed to predict antibody-antigen interactions and affinity without relying on crystal structures, facilitating structural inference [163]. Generative models employing backbone modeling and inverse folding techniques focus on predicting CDR structures, with some approaches exploring full-atom modeling [290, 300, 302, 307]. Additionally, diffusion probabilistic models integrated with equivariant neural networks have been introduced to co-design antibody sequences and structures, addressing challenges such as linking CDR sequences to 3D conformations and modeling their distribution within full antibody sequences [250].

#### CDR sequence optimization for affinity enhancement

Various DL techniques have been employed to enhance CDR sequence affinity. An LSTM-based approach was used to identify binding sites in DNA-binding hydrolytic antibodies (abzymes) from FASTA sequences [227], while comparative studies revealed that CNNs outperformed LSTMs in binding prediction. However, LSTMs provided valuable insights into subsequences correlated with known binding sites, demonstrating their utility in primary sequence analysis [227]. A transformer model, fine-tuned on 100,000 antibody sequences, was used for clustering clones, while a GAN model generated novel sequences, improving diversity and affinity [193]. A study using DGMs for de novo HER2 antibody design identified binders from a library of ~ 10⁶ HCDR variants, achieving binding rates of 10.6% for HCDR3 and 1.8% for HCDR1 [142]. Surface plasmon resonance (SPR) analysis of 421 binders found 71 with low nanomolar affinities, comparable to trastuzumab, and 11 high-affinity binders with equal or superior functionality, enhanced developability, and potency [142]. This approach has the potential to accelerate therapeutic antibody development for diverse targets.

Optimal CDR (OptCDR) generates CDR libraries with high antigen affinity while maintaining compatibility with humanization protocols and natural frameworks, demonstrating binding performance comparable to experimentally evaluated natural antibodies [80]. OptMAVEn enables de novo design of variable regions, enhancing binding affinity and reducing immunogenicity, with engineered CDRs showing high specificity and sensitivity for target epitopes [140]. The quality of the synthetic antibody libraries is vital for isolating effective recombinant antibodies [308]. Ens-Grad, an ML method, designs CDRs for human IgG antibodies and has shown improved target affinities over traditional phage display in experimental benchmarks [295]. By merging models from various experiments, it predicts effective antibody binding from high-throughput data without detailed structural information, enabling novel therapeutic development [295]. A DGM using long short-term memory (LSTM) was introduced for antibody sequence generation and prioritization, enabling the discovery of high-affinity antibody sequences [226]. These models demonstrate the ability to effectively model complex amino acid interactions critical for precise antigen recognition and binding, offering advantages over some conventional algorithms [149]. The LSTM model efficiently generates and prioritizes sequences, correlates likelihood with binding affinity, and monitors sequence enrichment, reducing repetitive mutation experiments and screening costs [149]. By leveraging next-generation sequencing (NGS) data, it optimizes CDR sequences, explores virtual libraries beyond phage display, and identifies key residues from limited data, enhancing high-affinity antibody discovery with fewer iterations [226].

GANs have been applied to generate functional protein sequences, addressing the randomness and low success rates of traditional GANs [85]. To optimize antibodies, a language-model-guided GAN (AbGAN-LMG) was developed, leveraging language models to improve GAN performance. AbGAN-LMG contributed to antibody optimization by generating diverse candidates and improving efficiency in design processes. In evaluations for COVID-19 and MERS, over 50% of sequences generated for AZD-8895 showed better developability than the original, and molecular docking identified 70 antibodies with higher affinity for the SARS-CoV-2 receptor-binding domain [85]. A convolutional neural network encodes antibody light and heavy chain CDR3s as images to distinguish binders from non-binders [223]. It employs in silico mutagenesis to identify critical CDR3 residues and generative adversarial networks to create synthetic antibodies targeting PD-1 and CTLA-4, as well as variable-length CDR3 sequences [223]. This study demonstrates the potential of DL to uncover patterns in antibody sequences, enhancing engineering, optimization, and discovery. IgDiff addresses antibody design challenges by generating highly designable antibodies with novel binding regions and well-aligned backbone dihedral angles to ensure structural integrity. It has shown strong performance in generating CDRs and pairing light and heavy chains, performing competitively with state-of-the-art models in benchmark evaluations [294]. A recent study introduced the Multi-channel Equivariant Attention Network (MEAN), which frames antibody design as a conditional graph translation problem. Using E(3)-equivariant message passing and a novel attention mechanism, MEAN demonstrated a 23% improvement in antigen-binding CDR design and a 34% boost in affinity optimization in benchmark evaluations [263].

#### Generating CDR libraries

AI-driven generative models have contributed significantly to creating diverse CDR libraries for antibody discovery. DGMs trained on extensive antibody sequence datasets have successfully designed high-affinity, epitope-specific antibodies, with some models demonstrating improved binding properties beyond those observed in training datasets [231]. A study utilizing a FACS-enriched yeast library from an immunized alpaca (*Lama pacos*) identified 104 sequences, yielding 103 unique single-domain antibodies (sdAbs) via next-generation sequencing [193]. The GAN model contributed by generating a virtual library to enhance CDR sequence diversity, potentially enabling a broader range of affinities and functions. Additionally, a lattice-based simulation framework was employed to evaluate ML-generated antibody sequences by simulating 3D structures from 1D sequences, further validating the feasibility of high-throughput antibody design [231]. OptCDR directly creates diverse CDR libraries tailored for high-affinity binding, broadening the screening space while maintaining strong binding properties in selected candidates [80]. The Immunoglobulin Language Model (IgLM), a DGM trained on 558 million antibody sequences, was recently developed. Using a text-infilling approach with bidirectional context, IgLM generates variable-length antibody sequences, enabling full-length antibody design across species. It creates CDR loop libraries with improved in silico developability, with optimizations aimed at reducing solubility issues, aggregation, and immunogenicity [215]. ReprogBERT, a novel approach using Model Reprogramming, adapts a pretrained English language model for protein sequence infilling. In benchmark evaluations, it generated highly diverse CDR sequences, showing up to twice the diversity of baseline models while maintaining structural integrity and naturalness [211]. GAN-based approaches, such as AbGAN-LMG and CNN-based mutagenesis, can expand candidate CDR sets for multiple targets, contributing to the generation of synthetic libraries that support antibody discovery workflows [85]. Computational protein design seeks to create novel, diverse protein sequences for a given structure, but it remains challenging. A recent study benchmarked three DGMs: the autoregressive model (AR), the graph neural network (GVP), and Fold2Seq. Fold2Seq generated diverse antibody sequences while maintaining structural integrity, demonstrating superior performance over other models in benchmark comparisons [254].

#### Optimizing CDR immunogenicity

To address immunogenicity concerns in antibody design, AI models have been developed to aid in immunogenicity prediction and optimization, demonstrating promising results in various benchmarks. EquiPocket, an E(3)-equivariant geometric graph neural network, was introduced to predict ligand-binding sites, effectively capturing irregular protein structures and surfaces, which can contribute to immunogenicity assessment [249]. Modeling sequence variation effects on protein function is vital for protein design [199]. Evolution encodes functional information in protein sequences, allowing unsupervised models to predict variant effects [199]. Protein language models utilizing zero-shot inference have shown strong performance in predicting the functional impacts of sequence variations, providing insights into immunogenicity assessment without requiring additional training or experimental data [199]. Further advancements in synthetic antibody research, supported by deep sequencing and advanced computational algorithms, have expanded antibody repertoire analysis, facilitated novel sequence prediction, and enabled de novo antibody generation, contributing to progress in immunology and biological therapeutics [23]. OptMAVEn’s de novo design strategy aims to reduce immunogenicity by emphasizing human-like sequences and minimizing T-cell epitopes, while maintaining high specificity and sensitivity for selected antigens [140]. Many generative models, such as IgLM or AbGAN-LMG, incorporate developability filters or scoring metrics to help address immunogenic risks, aiming to design candidate CDRs with reduced reactivity and closer alignment to human germline frameworks [85, 215]. OptCDR’s ‘standard humanization’ approach similarly focuses on mitigating potential immune responses by reducing non-human elements in designed CDR sequences, improving compatibility with human frameworks.

#### Other antibody engineering methods

Nach0, a multi-domain, multi-task encoder-decoder model pre-trained on datasets like scientific literature, patents, and molecule strings, was fine-tuned with the NeMo framework. It demonstrated competitive performance against state-of-the-art models in single- and cross-domain tasks, producing high-quality molecular and textual outputs [309]. An RNN trained on ~ 24 million UniRef50 sequences improved protein function prediction, addressing the challenge of generalizing predictions to evolutionarily distant sequences. These advances support protein engineering by aiding the identification and prioritization of functional sequences, contributing to efforts in optimizing protein diversity and function [304]. Diffusion probabilistic models and equivariant neural networks, including AbDiffuser, offer alternatives or complements to purely physics-based or purely data-driven approaches by modeling sequence and structure simultaneously, with the potential for improved efficiency [290]. Probabilistic methods, including directed search algorithms, have been applied to de novo antibody design, helping identify sequences with desired traits. Directed searches identify sequences with specific characteristics, while probabilistic approaches estimate site-specific amino acid probabilities to achieve target structures [310]. This approach supports de novo protein design and combinatorial library engineering by converting probabilities into nucleotide distributions, helping DNA synthesizers generate degenerate sequences with improved fidelity [310]. Designing diverse, stable, and well-expressed antibody libraries is challenging, as large synthetic libraries often contain sequences with reduced functionality [310]. To address this, advanced NLP-based computational methods were developed for alignment-free prediction and functional sequence design [253]. DGMs predicted missense and indel effects and were applied to design a 105-nanobody library, which demonstrated improved expression compared to larger synthetic libraries, contributing to protein design and biotherapeutic research [253]. A separate study combined phylogenetic and atomistic calculations to optimize protein stability, expressibility, and activity, offering evolutionary insights into enzymes and binders [311]. Table 3 summarizes AI-based computational studies, their applications, and methods, offering insights into addressed challenges and solutions.

**Table 3:** Overview of selected AI-based computational studies: applications and insights

S. No	Year	Problem Addressed	Algorithms Used	Description	Key Results	Main Conclusion	References
1	1999	Antibody engineering, targeted mutations	X-PLOR and REFMAC for structure optimization, Surface Plasmon Resonance (SPR) for kinetic analysis, Structural modeling using Brookhaven Protein Data Bank (1bj1)	Enhanced the affinity and potency of Fab-12 targeting VEGF for cancer treatment. Targeted mutations in heavy-chain CDRs improved binding affinity, with CDR-H2 and CDR-H3 having the most significant impact	Y0243-1 variant (CDR-H1) showed a threefold affinity increase, while Y0317 (CDR-H3) achieved a 20-fold improvement. Final variant Y0317, with six mutations, resulted in a 100-fold increase in potency. Structural analysis and X-ray crystallography confirmed improved binding interactions	CDR mutations and phage display significantly enhanced Fab-12 affinity for VEGF, demonstrating the role of targeted mutations and computational modeling in antibody engineering	[283]
2	1987	Understanding canonical structures in immunoglobulins	Structural analysis of immunoglobulins, Sequence-structure correlation mapping	Linked immunoglobulin amino acid sequences to 3D structures of antigen-binding sites, identifying key residues that shape hypervariable regions crucial for antigen binding	Identified key residues in hypervariable regions and β-sheet frameworks dictating main-chain conformations. Revealed that many immunoglobulins share predictable structural patterns, categorized as 'canonical structures.' Demonstrated that structural predictions based on sequence data improve antibody modeling and engineering	Canonical structures provide a framework for predicting antibody structures from sequences, enhancing the accuracy of antigen-binding site modeling for therapeutic applications	[312]
3	2000	Antibody modeling	WAM (Web Antibody Modelling) algorithm, CONGEN conformational search, Energy screening via Eureka (a solvent-modified VFF), Clustering using RMS deviation	Developed WAM, an improved antibody modeling algorithm focusing on variable regions (Fv) and CDRs, particularly enhancing CDR-H3 loop modeling using canonical class concepts and energy screening methods	Achieved higher accuracy (1.7–2.0 Å rmsd compared to traditional methods. Energy screening using a solvent-modified VFF (Eureka) improved structural refinement. Statistical analysis showed rmsd values as low as 1.3 Å for short loops and up to 2.0 Å for longer loops	WAM enhances antibody structure prediction accuracy through knowledge-based screening and energy optimization, offering an advanced modeling approach accessible online	[313]
4	2006	Affinity improvement of antibodies	Structure-based computational design, Side chain repacking, Electrostatic optimization, Energy evaluation techniques	Optimized antibody binding affinity to the I-domain of integrin VLA1 using computational modeling, refining amino acid positioning and electrostatic interactions to enhance affinity. Experimental validation included competition ELISA and KinExA assays	Created a quadruple mutant with tenfold increased affinity to the VLA1 integrin I-domain. Hit rate analysis (12% success from 83 mutants) and EC50 comparisons quantified improvements. Crystal structures confirmed hydrogen bonding's role in high-affinity mutations	Structure-based computational design significantly enhances antibody binding affinity, demonstrating the role of electrostatic optimization and iterative modeling in therapeutic antibody development	[162]
5	2007	Comparison of B-cell epitope prediction methods	Scale-based (DiscoTope, PIER, ProMate, ConSurf), Patch-based (CEP, PPI-PRED, ClusPro (DOT), PatchDock)	Evaluation of eight web servers for B-cell epitope prediction using probability scores and docking-based interface identification	PatchDock achieved the highest AUC (> 0.69) and sensitivity (> 75%). ProMate and PPI-PRED showed moderate performance, with ProMate slightly outperforming PPI-PRED. ConSurf and DiscoTope exhibited poor performance (AUC ~ 0.6), while ClusPro (DOT) and CEP performed near random	PatchDock was the most effective method, while others had limited success	[306]
6	2007	Improving antibody affinity	Hierarchical computational design, A* search and Dead-end elimination, Poisson-Boltzmann electrostatics	Developed a computational method to enhance antibody affinity by optimizing electrostatic interactions, evaluating single mutations systematically, and incorporating experimental feedback for iterative refinement	Achieved a tenfold affinity increase in cetuximab and a 140-fold improvement in D44.1. Identified beneficial mutations in bevacizumab and 4–4–20. Demonstrated a 460% success rate in predicting beneficial single mutations, with D44.1 improving to 30 pM and cetuximab to 52 pM	Iterative computational design significantly enhances antibody affinity, demonstrating the effectiveness of computational methods in optimizing protein binding affinity for therapeutic applications	[281]
7	2008	Identification of CDRs and B-cell epitope characteristics	Automated structure-based method leveraging antibody-protein complexes	Developed an automated method to accurately identify CDRs and B-cell epitopes by analyzing structural regions from known antibody-protein complexes, overcoming sequence-based tool limitations	CDRs exhibit a highly restricted composition dominated by four amino acids, while aliphatic-hydrophobic residues (A, I, L, V) are underrepresented. Histidine uniquely maintains consistent interactions across different molecular contexts	Structural biology and automation improve CDR identification, enhancing insights into antibody-antigen interactions	[267]
8	2008	Antibody loop replacement study	Rosetta software for structural modeling and hydrogen bond calculations, High-throughput screening, Tailored DNA libraries	Explored CDR loop length modifications to enhance antigen binding and improve affinity. Tested whether longer CDR loops could create new interactions with the VLA1 antigen, expanding antibody design possibilities	Affinity comparisons showed over 100-fold lower binding in modified constructs compared to the wild-type antibody. Replacing the L1 loop in an anti-VLA1 antibody did not improve affinity due to structural instability caused by domain swapping. A second round of modifications (Leu51 to Ser mutation) stabilized the loop and eliminated dimerization	Structural instability limited the success of CDR loop replacements, but targeted mutations stabilized the loop, highlighting the need for careful loop engineering in antibody design	[286]
9	2008	Antibody affinity maturation	In silico modeling, Molecular dynamics, Docking, CDR walking mutagenesis (targeting CDR-H3 and CDR-L3)	Optimized the affinity of human anti-gastrin TA4 scFv for therapeutic use. Combined computational modeling with experimental validation, using phage display technology, CDR walking mutagenesis, and molecular dynamics to assess stability and key contact residues	Achieved a 454-fold affinity improvement (K_D_ = 13.2 nM). Targeted mutagenesis focused on CDR-H3 and CDR-L3 to reduce antigenicity and immunogenicity. Structural modeling guided rational design, demonstrating the effectiveness of computational methods	The integration of computational modeling and experimental approaches effectively accelerates high-affinity therapeutic antibody development, demonstrating the potential of structure-based design	[285]
10	2008	Antibody homology modeling and antibody-antigen docking	RosettaAntibody protocol, Monte Carlo minimization, Rigid-body optimization, Comparative modeling, De novo CDR H3 modeling, Ensemble docking predictions	Developed RosettaAntibody, a high-resolution homology modeling protocol for antibody variable regions (Fv). Combines canonical CDR loop modeling, de novo CDR H3 predictions, and V_L_-V_H_ orientation optimization to improve structure-based applications	Achieved a median RMSD of 1.5 Å for antigen-binding pockets. Demonstrated moderate to high docking accuracy for 7 of 15 targets. Statistical validation via rmsd benchmarking showed many models aligned within 2.0 Å of crystal structures	RosettaAntibody improves antibody homology modeling and docking accuracy, providing valuable insights for computational docking, therapeutic antibody engineering, and protein design	[152]
11	2010	CDR design	OptCDR (De novo CDR generation), Structural refinement, Mutation prediction, Computational selection for affinity optimization	Developed OptCDR, a computational framework for de novo CDR generation, optimizing binding affinity and specificity. Unlike traditional methods modifying existing antibodies, OptCDR mimics natural evolution to identify favorable mutations, streamlining iterative refinement and accelerating antibody discovery	Tested on three antigens (fluorescein, hepatitis C capsid peptide, and VEGF), demonstrating high-affinity antibody design across diverse targets. Employed statistical scoring functions for binding optimization and computational benchmarking against known binders	OptCDR efficiently generates diverse antibody libraries with enhanced specificity and affinity, proving its value as a computational alternative to time-consuming experimental approaches	[80]
12	2012	Structural analysis of B-cell epitopes	Hobohm2 algorithm for redundancy removal, bootstrapping for statistical validation	Used interaction vectors and amino acid pair frequencies to define antigen–antibody similarity, applying bootstrapping to assess statistical distributions for epitope composition trends	B-cell epitopes are flat, oval-shaped, and align with antibody binding sites at −30Â° to 60Â°. Typically consist of ~ 15 residues with a hydrophobic core for stability and charged edges for interaction. Hydrophilic residues dominate the binding interface, while positively charged residues are underrepresented	B-cell epitopes differ from general protein–protein interfaces, necessitating specialized prediction models for improved immunology and vaccine design	[314]
13	2013	Antibody library and optimization of CDR	Predator synthetic antibody library, Targeted randomization, Trinucleotide synthesis, Hydrophilic mutation selection	Developed Predator, a synthetic antibody library designed to improve folding, reduce aggregation, and enhance functional antibody selection. Features a high diversity (6.2 × 10⁷ clones) with optimized CDR design for increased binding efficiency	Predator mimics the human immune response by designing CDR3 compositions based on functional human antibodies. Incorporates targeted randomization at seven positions, omitting suboptimal amino acids while optimizing for stability and solubility. Phage display selection yielded specific antigen binders	Predator is an effective synthetic antibody library built on an aggregation-resistant HEL4 scaffold, allowing modular cloning and affinity maturation for improved therapeutic antibody development	[308]
14	2014	Prediction of antibody-specific B-cell epitopes	Custom computational framework using residue-pairing and interface characteristics	Developed a method utilizing antibody sequences to identify discontinuous epitopes on antigens, emphasizing residue-pairing and interface characteristics	A dataset of 646 Ab-Ag structures from PDB was compiled, and antibody sequences were clustered using BLASTCLUST (≥ 97% identity). Computational predictions refined through Patch-per-Ab and Patch-per-group approaches improved accuracy in D8 antigen analysis. Validation techniques included X-ray crystallography, peptide ELISA, deuterium exchange, site-directed mutagenesis, and cross-blocking	Integrating antibody-specific predictions with cross-blocking experiments enhances precision in identifying overlapping epitopes, confirming residues within conformational B-cell epitopes	[315]
15	2014	Antibody generation and immunogenicity minimization	OptMAVEn (de novo modeling), Mixed-Integer Linear Programming (MILP), Iterative Protein Redesign & Optimization (IPRO), MHC/T-cell epitope quantification (HSC score)	Developed OptMAVEn, a computational method for designing antibody variable regions with optimized affinity and reduced immunogenicity, eliminating reliance on existing antibodies or immunized animals. Enhances binding affinity using biophysical models and a humanization procedure to minimize immunogenicity	OptMAVEn efficiently positioned antigens, rediscovered native structures, and mimicked natural evolution to improve affinity. Validated against targets like influenza hemagglutinin and HIV gp120, ensuring lower immune recognition through a 9-mer approach. Modular Antibody Parts (MAPs) database selected components for high affinity and low immunogenicity	OptMAVEn extends OptCDR for de novo antibody design, integrating humanization strategies to minimize immune response, accelerating therapeutic and vaccine antibody development	[140]
16	2014	Antibody design with elongated CDRs	Scaffold-based antibody engineering, Computational modeling, X-ray crystallography, Flow cytometry, Tag-lite HTRF binding assays	Engineered antibodies with elongated CDRs, particularly CDRH3 from bovine antibodies (BLV1H12), to enhance binding affinity and access hidden ligand sites, expanding therapeutic potential	Modified CXCR4-binding peptides were grafted into CDRH3 and CDRH2, optimizing binding without steric hindrance. Engineered antibodies (bAb-AC1, bAb-AC2, bAb-AC3) bound CXCR4-expressing cells with high affinity (Kd = 2.1–19.8 nM), blocking SDF-1-induced CXCR4 activation and inhibiting cell migration	Scaffold-based antibody engineering using elongated CDRs provides high specificity and therapeutic potential for targeting CXCR4-related diseases, including cancer and HIV	[316]
17	2017	All-Atom Energy Function for Macromolecular Modeling and Design	Rosetta Energy Function (REF15), van der Waals interactions, Electrostatics, Torsional energy terms, PyRosetta	Developed REF15, an enhanced energy function for the Rosetta suite, improving biomolecular modeling accuracy. Originally designed for proteins, it now supports RNA, DNA, carbohydrates, and synthetic macromolecules	REF15 refines structural insights by optimizing energy calculations, improving computational speed, and expanding applications in drug discovery, synthetic biology, and nanomaterial design	REF15 significantly enhances Rosetta's capabilities in biomolecular modeling, strengthening its role in structural biology, vaccine development, and therapeutic engineering	[317]
18	2017	Computational design of stable and functional binding antibodies	AbDesign algorithm, Rosetta design calculations, Statistical modeling based on antibody datasets	Developed a computational framework for designing stable and functional Fvs, addressing structural challenges such as large loops and buried polar networks. Used sequence constraints from natural antibodies to maintain backbone integrity and enhance stability	Engineered Fvs achieved mid-nanomolar affinities and stability comparable to natural antibodies. Iterative design and experimentation refined structures by correcting flaws like unpaired charges and cavities. Crystallography confirmed atomic accuracy of designed Fvs, supporting precise antibody engineering	The study established principles for designing stable, functional antibodies, demonstrating that iterative computational design and sequence constraints enable high-affinity, structurally stable antibody fragments	[284]
19	2019	Protein Engineering using Generative Models	Score Matching, Pseudolikelihood, Loopy Belief Propagation, Stochastic Backpropagation, Generative Adversarial Nets, Deep AutoRegressive Networks, Reweighted Wake-Sleep	Protein design using generative models, improving sequence-structure consistency and predicting novel functional variants for efficient protein engineering	Enhanced structural accuracy and sequence variability, improving protein folding predictions	Generative models significantly improve protein design, paving the way for more reliable synthetic protein development	[318]
20	2019	Generative models in protein design	Graph-based conditional generative models, Inverse protein folding, Structural generalization, Graph encodings	Developed a generative framework for protein sequence design to solve the inverse folding problem, ensuring sequences fold into specific 3D structures. This is critical for biomedicine, energy, and materials science applications	Graph-based models capture long-range dependencies, improving structural generalization. Outperformed Rosetta in sequence accuracy and computational efficiency, reducing trial-and-error in protein design	Graph-based conditional generative models significantly advance targeted biomolecule design, enhancing speed, reliability, and efficiency in protein engineering	[319]
21	2020	Prediction of antigen–antibody binding interfaces	Graph Convolutional Networks (GCNs), Attention Mechanism, Transfer Learning	Developed computational methods to predict antibody-antigen binding interfaces, enhancing drug and vaccine design while addressing limitations of experimental approaches using large datasets	Conv2-layer with Attention Layer achieved the highest performance (AUC-PR = 0.8), while Convolution Alone performed significantly worse (AUC-PR = 0.48). Attention-enhanced models consistently outperformed others, demonstrating the effectiveness of combining graph convolutions with attention mechanisms	A DL framework using graph convolutions, an attention layer, and transfer learning achieved state-of-the-art accuracy, improving antibody-antigen interaction predictions while offering interpretable insights	[297]
22	2020	CDR design	Ens-Grad framework, CNNs, Gradient ascent optimization, Ensemble learning, Argmax transformation	Developed Ens-Grad, a ML-based approach for designing high-affinity CDRs in human IgG antibodies, eliminating off-target effects without requiring detailed target structures. Integrates experimental data to enhance specificity and optimize antibody sequences	Trained on phage display panning data, linking CDR-H3 sequences to binding affinity. Achieved AUROC of 0.960 in predicting ranibizumab enrichment, demonstrating high accuracy in distinguishing binding vs. non-binding sequences. Ensemble learning with voting-thresholding strategy improved diversity and robustness	Ens-Grad provides a modular, ML-driven approach for precise antibody design, demonstrating the effectiveness of ensemble learning in optimizing high-affinity candidates	[295]
23	2020	CDR H3 loop structure prediction	DeepH3 (Deep residual neural network—ResNet), Geometric potential refinement, Distance and orientation-based learning	Developed DeepH3, a DL-based approach to predict CDR H3 loop conformations, converting sequence data into geometric potentials to refine RosettaAntibody-generated models	DeepH3 outperformed RosettaAntibody in 33 of 49 targets, reducing RMSD by 32.1% (1.4 Å improvement). Achieved an average RMSD of 2.2 ± 1.1 Å (top 1) and 1.9 ± 0.9 Å (top 5), demonstrating superior predictive accuracy	DeepH3 significantly enhances CDR H3 loop modeling accuracy using DL-based distance and orientation potential refinements, making it a valuable tool in antibody structural predictions	[148]
24	2020	Denoising Diffusion Probabilistic Model	Diffusion probabilistic models, Sampling algorithm, Progressive lossy compression, Reweighting objective algorithm	Developed a diffusion-based generative model for high-quality image synthesis, leveraging noise-adding and denoising processes inspired by nonequilibrium thermodynamics	Achieved an Inception Score of 9.46 and FID of 3.17 on CIFAR10, outperforming many existing generative models. Demonstrated superior sample fidelity, distinguishing it from GANs and VAEs	Diffusion probabilistic models provide an efficient and effective approach to image synthesis, achieving state-of-the-art results in generative modeling	[320]
25	2021	Paratope-epitope prediction	Para-EPMP (CNNs + GNNs for localized paratope prediction), Epi-EPMP (Graph Attention Networks + Multi-task Learning for scattered epitopes)	Developed EPMP architecture, combining Para-EPMP for paratope prediction and Epi-EPMP for epitope prediction. Para-EPMP models antigen-dependent paratope interactions, while Epi-EPMP handles scattered epitopes using graph-based structural learning	Para-EPMP achieved the highest paratope prediction performance (AUC ROC = 0.966, AUC PR = 0.752), outperforming PECAN and other models. Epi-EPMP led epitope prediction with AUC ROC = 0.710, AUC PR = 0.277, surpassing alternative approaches	EPMP architecture effectively models paratope-epitope interactions by integrating both sequence and structural data, enhancing antibody-antigen interaction predictions and setting new benchmarks in epitope and paratope modeling	[299]
26	2021	Virtual screening of antibodies	DLAB framework (Deep Learning for Antibody Binding), DLAB-VS (CNN-based binding classifier), DLAB-Re (Pose refinement), Ensemble Learning	Introduced DLAB, a DL framework for structure-based antibody screening, enhancing virtual screening without prior binders. DLAB-VS classifies binders/non-binders using structural features, while DLAB-Re refines pose selection based on high-scoring inputs (fnat > 0.7 binders, < 0.1 non-binders). Post-snapshot evaluation ensures generalization to unseen targets, optimizing docking accuracy	DLAB-VS, combined with ZDock outputs, achieved superior AUC scores, enhancing antibody ranking and therapeutic candidate identification. CNNs were used for binding classification, trained on high-quality docking poses with data augmentation to improve generalization	DLAB enhances antibody-antigen binding prediction, improving docking accuracy and virtual screening. By integrating CNN-based binding classification and optimized pose refinement, it generalizes across low-identity targets and antigen variants, streamlining therapeutic antibody development	[296]
27	2021	Antibody design and affinity maturation	Long Short-Term Memory (LSTM) networks, Phage display panning, Next-generation sequencing (NGS), Likelihood scoring (Negative Log-Likelihood—NLL)	Developed an LSTM-based framework for antibody sequence generation and binding affinity prediction, reducing reliance on traditional labor-intensive mutation experiments. Uses NGS data to generate high-affinity variants while avoiding combinatorial complexity	Generated sequences with 1800-fold higher affinity than the parental clone. Likelihood scores (NLL) correlated with actual binding affinity, improving candidate selection. Cross-validation confirmed the robustness of LSTM over traditional frequency-based screening	LSTM-based affinity maturation streamlines antibody discovery by efficiently prioritizing high-affinity candidates, reducing cost and labor while outperforming traditional methods	[226]
28	2021	Antibody design and affinity maturation	Long Short-Term Memory (LSTM) networks, Graph Neural Networks (GNNs), Hag-Net (graph-based network for antibody-antigen interactions), Pairwise prediction strategy	Developed sequence-based DL models to optimize therapeutic antibody leads, predicting antibody-antigen interactions and binding affinities without requiring crystal structures. Pairwise prediction strategy improves affinity assessment by comparing closely related mutants	Hag-Net achieved an AUC > 0.90 in five-fold cross-validation and 0.70 in out-of-distribution tests. Graph Neural Networks improved structural learning, enhancing binding affinity predictions over conventional in silico approaches	DL-based models, including LSTM and GNNs, provide computationally efficient, scalable, and accurate affinity predictions, enabling broader applicability in industrial antibody development	[163]
29	2021	Benchmarking of models used in antibody sequence design	Autoregressive model (AR), Geometric Vector Perceptron (GVP), Fold2Seq (Transformer-based model)	Benchmarked three deep generative models (DGMs) for designing diverse antibody sequences while maintaining structural constraints, addressing challenges in protein design	Fold2Seq achieved the best balance between sequence diversity and structural integrity. Evaluations based on sequence diversity, structural accuracy (TM-score, RMSD), and physicochemical properties confirmed its superior performance	Fold2Seq excels in generating structurally consistent and diverse antibody sequences, highlighting the potential of DGMs in therapeutic antibody design	[254]
30	2021	Score-based generative modeling using SDEs	Stochastic Differential Equations (SDEs), Score-based generative modeling, Predictor–Corrector (PC) framework, Neural ODEs	Developed a generative modeling framework that transforms noise into complex data distributions, enhancing sample generation and solving inverse problems in data science	Achieved state-of-the-art performance on CIFAR-10 with an Inception Score of 9.89 and an FID of 2.20. Enabled conditional generation, image inpainting, and colorization without retraining, expanding practical applications	SDE-based generative modeling with predictor–corrector frameworks enhances sampling efficiency, improves accuracy, and sets new benchmarks in high-fidelity image synthesis	[321]
31	2021	Protein structure prediction	Two-Track Network, Three-Track Network, Attention Mechanisms, End-to-End Learning, SE(3)-Equivariant Transformer, pyRosetta, Crop Training Method	DeepMind achieved accurate predictions at CASP14 with a three-track network architecture. This network integrates information from the 1D sequence, 2D distance map, and 3D coordinates, and transforms and integrates it successively to achieve the best performance	The three-track neural network integrates 1D sequences, 2D distance maps, and 3D atomic coordinates for accurate protein structure prediction. It uses attention mechanisms for long-range interactions and SE(3)-equivariant layers for spatial consistency. End-to-end training optimizes predictions, with PyRosetta refining atomic models. RoseTTAFold is benchmarked against AlphaFold2 and trRosetta using TM-score, Cα-RMSD, and RMS error	This method matched DeepMind's CASP14 results, solved challenging modeling problems, provided insights into unknown protein functions, and quickly generated accurate protein–protein complex models from sequences. It is available to the scientific community to advance protein structure predictions and biological research	[322]
32	2021	Affinity Maturation using AntiBERTy Model	AntiBERTy, Multi-head attention pooling, Deep multiple instance learning (MIL)	AntiBERTy uses 558 million antibody sequences to model affinity maturation, revealing evolutionary pathways and key binding residues for therapeutic design	Improved clustering of antibody sequences into evolutionary trajectories, enhancing antigen-specific affinity maturation studies	AntiBERTy provides deep insights into antibody evolution, assisting vaccine and therapeutic antibody development	[241]
33	2021	Molecular Conformation Generation using ConfGF	Torsionnet, MARS (Markov molecular sampling), Conditional Variational Graph Auto-Encoder (CVGAE), Graphaf, Deep Potential Molecular Dynamics (DPMD), SchNet, Neural Message Passing, SE(3)-Transformers	ConfGF enhances molecular conformation generation by predicting stable 3D structures from 2D graphs using Langevin dynamics for more accurate results	Achieves 88.49% coverage and a 0.2673 Å MAE on QM9, surpassing existing molecular modeling methods	ConfGF generates diverse and accurate molecular conformations, improving molecular modeling for drug discovery	[323]
34	2021	Categorical Distributions with Argmax Flows and Multinomial Diffusion	Argmax Flows, Multinomial Diffusion, Importance Weighted Autoencoders, Real NVP (Real-valued Non-Volume Preserving), NICE (Non-linear Independent Components Estimation)	Introduces Argmax Flows and Multinomial Diffusion to improve categorical generative models, ensuring better coherence in high-dimensional discrete data	Outperforms traditional dequantization methods in negative log-likelihood, achieving high accuracy in text generation and image segmentation	Argmax Flows and Multinomial Diffusion significantly enhance categorical data modeling, improving text and image generation tasks	[324]
35	2021	Protein Structure Prediction using AlphaFold	AlphaFold, TensorFlow, Sonnet, JAX, Haiku, XLA compiler	AlphaFold revolutionizes protein structure prediction by integrating DL with physical constraints, achieving atomic accuracy on CASP14 challenges	Achieves atomic accuracy, surpassing conventional methods in protein structure modeling	AlphaFold sets new standards for computational biology, making protein structure prediction highly accurate and accessible	[28]
36	2021	Protein Structure Prediction using Deep Residual Networks	RaptorX-Contact, AlphaFold, Residual Neural Networks, PyRosetta, TensorFlow	Deep residual networks enhance protein structure predictions by incorporating inter-residue distances and orientations, outperforming previous methods	TM-score of 0.625, outperforming Robetta and achieving superior predictive accuracy	Inter-residue distance prediction advances protein modeling accuracy, leading to better structure-based drug design	[325]
37	2021	Antibody Structure Prediction using DeepAb	DeepAb, Focal Loss (for calibrating deep neural networks), MUFOLD (for protein 3D structure prediction), Rosetta3 (for macromolecular modeling and design)	DeepAb uses DL to predict antibody structures, outperforming RosettaAntibody. It combines a residual convolutional network and biLSTM-based RNN to learn inter-residue distances and orientations from sequences. The model refines predictions and generates 3D structures using Rosetta, advancing therapeutic antibody development	Improved RMSD accuracy by 14%−18% for heavy chains and 16%−17% for light chains	DeepAb enhances antibody prediction, advancing drug discovery and therapeutic antibody optimization	[88]
38	2021	Protein Design and Variant Prediction	Deep Generative Models, Autoregressive Models, Variational Autoencoders (VAE) (as a reference point for comparison), Probabilistic Models (such as EVmutation)	A deep generative model (DGM) using natural language processing (NLP) was developed to predict and design functional proteins without sequence alignments. It effectively handles highly variable sequences, such as antibodies and nanobodies	The model was tested on a designed nanobody library, yielding 1.5 × higher expression and nearly doubled mean display levels (166,193 vs. 92,183 units). Mutation effect predictions achieved an AUC of 0.90	An alignment-free DGM that designs high-expression nanobody libraries and predicts protein sequences accurately. It significantly improves computational protein engineering and therapeutic antibody development	[253]
39	2021	Mutations Effect on Protein Function	ESM-1v, MSA Transformer, EVMutation, DeepSequence JackHMMer	Zero-shot inference enables efficient protein function prediction without specialized training, reducing data dependency and computation. It accelerates research, uncovers novel functional relationships, and advances scalable protein engineering	Deep mutational scanning uses computational models to predict mutation effects on proteins. Supervised methods (e.g., regression, random forests) offer interpretability, while ensemble models (Revel, CADD) improve accuracy. Language models (UniRep, ESM) capture sequence patterns, and unsupervised tools (SIFT, EVMutation) leverage evolutionary data. Benchmarking ensures accuracy using Spearman correlation (ρ) and cross-validation. Zero-shot inference enhances generalizability, driving advances in protein engineering and functional analysis	Researchers have shown that protein language models can use zero-shot inference to predict the functional effects of sequence variation, without experimental data or additional training. This method leverages evolutionary patterns in protein sequences, providing an efficient and generalized solution for predicting protein functions from sequence data	[199]
40	2021	Denoising Diffusion Models in Discrete State-Spaces	Absorbing-state diffusion, Nearest-neighbor (NN) diffusion, Uniform diffusion, Cosine scheduling, Mutual information scheduling	D3PMs enhance discrete data modeling by introducing multinomial diffusion for structured transition matrices. Benchmarked on CIFAR-10, they outperform traditional dequantization methods in text and image synthesis, improving log-likelihood, sample quality, and generative accuracy	D3PMs achieved an Inception Score (IS) of 11.47 and FID ≤ 2.94. on CIFAR-10, surpassing continuous-space DDPMs in text and image generation. Their structured transition matrices improved categorical data modeling	D3PMs refine generative models for categorical data, enhancing text and image synthesis efficiency while maintaining structured transition matrices	[326]
41	2022	Antibody affinity optimization and developability	AI and ML-driven sequence analysis, Next-generation sequencing (NGS), Bioinformatics-guided CDR design, Computational CDR-FWR shuffling	Leveraged NGS data from human antibody repertoires, including COVID-19 patient antibodies, to optimize CDR and framework selection for antigen recognition. Used AI and ML to improve affinity and developability more efficiently than traditional methods	Framework-CDR shuffling optimized CDR-framework combinations, preserving natural diversity while improving affinity and specificity against SARS-CoV-2 variants. CB79, derived from SARS-CoV-2-neutralizing antibody H4, exhibited a sevenfold affinity increase and 75-fold improved neutralization, effectively blocking viral entry	Computational CDR-FWR shuffling is an efficient strategy for antibody development, enhancing affinity, stability, and specificity for therapeutic applications against SARS-CoV-2 and other diseases	[264]
42	2022	B-cell conformational epitope prediction	Pretrained Protein Language Models (ESM-1v, ESM-IF1), Transfer Learning, Binary Classification, Ensemble Learning	Developed the SEMA model to enhance conformational B-cell epitope prediction, addressing limitations of current methods in vaccine and immunotherapy research. Tested on the SARS-CoV-2 receptor-binding domain (RBD), capturing structural and functional features for improved predictions	SEMA achieved high accuracy (ROC AUC 0.76), outperforming existing tools. Utilized sequence-based (SEMA-1D) and structure-based (SEMA-3D) approaches to improve interpretability and immunogenic potential assessment	SEMA, using transfer learning and pretrained DL models (ESM-1v, ESM-IF1), enhanced conformational B-cell epitope prediction and effectively ranked immunodominant regions in SARS-CoV-2 RBD	[237]
43	2022	Predicting antibody-antigen interactions	AbAgIntPre (Siamese-like CNN architecture), CKSAAP encoding for spatial amino acid relationship representation	Developed AbAgIntPre, a DL method using a Siamese-like CNN architecture to predict antibody-antigen interactions from amino acid sequences. Addresses limitations of traditional methods and 3D structure-dependent computational approaches	Achieved an AUC of 0.82, excelling in SARS-CoV datasets. Outperformed traditional ML models like Random Forest and SVM, demonstrating higher accuracy and reducing false positives	AbAgIntPre accelerates antibody screening and design by predicting antibody-antigen interactions from sequence data, complementing experimental methods with improved accuracy and efficiency	[157]
44	2022	Understanding protein binding in DNA-binding antibodies	Long Short-Term Memory (LSTM) networks, CNNs	Used large datasets to predict binding sites in autoimmune antibodies, advancing vaccine design and synthetic pharmacology for autoimmune disease treatments	CNNs achieved higher accuracy (96.56% for binding, 97.81% for non-binding) compared to LSTMs (87.07% and 88.56%). LSTMs captured long-range dependencies, enhancing interpretability, while CNNs efficiently detected local features	CNNs are more effective for direct binding prediction, while LSTMs provide deeper insights into binding mechanisms. A hybrid model could integrate both strengths for improved predictions in autoimmune antibody research	[227]
45	2022	Protein design	Diffusion models (DDPMs), SE(3)-equivariant frameworks, RFdiffusion, RoseTTAFold, Gaussian noise, Brownian motion	Developed a DL framework for de novo protein design, focusing on binder creation and symmetric architecture design. Improved upon constraints in backbone geometry and sequence-structure complexities	RFdiffusion generated diverse, biochemically precise protein structures, leveraging SE(3)-equivariant modeling and self-conditioning techniques. Experimental tests on hundreds of designs confirmed its reliability	RFdiffusion enhances de novo protein backbone design, excelling in monomers, binders, oligomers, enzyme scaffolds, and therapeutic proteins, advancing drug discovery and synthetic biology	[302]
46	2022	Antibody Backbone and Side-Chain Conformations Prediction	DeepSCAb (DL model), Inter-residue module, Attention mechanisms, Integration with RosettaAntibodyDesign	Developed DeepSCAb, a DL model predicting full F_V_ antibody structures, including backbone and side-chain conformations, directly from sequences, improving accuracy without prior structural input	DeepSCAb outperforms rotamer repacking methods, handling structural variability in solvent-exposed residues. It maintains accuracy across environments and integrates with antibody design tools for flexible and robust predictions	DeepSCAb enhances antibody modeling by predicting inter-residue geometries and side-chain dihedrals from sequence data, improving structure prediction and aiding therapeutic antibody design	[292]
47	2022	CDR Loop Structure Prediction	ABlooper, E(n)-Equivariant Graph Neural Networks (E(n)-EGNNs), Rotational and Translational Invariance, Parallel EGNNs	Developed ABlooper, a DL model that predicts CDR-H3 loop structures with high accuracy in under five seconds, outperforming traditional methods like ABodyBuilder, making it ideal for large-scale antibody research	ABlooper achieves an average RMSD of 2.49 Å, improving to 2.05 Å for high-confidence predictions, surpassing ABodyBuilder. It leverages five parallel EGNNs with four layers, minimizing RMSD and L1 loss for structural accuracy	ABlooper enhances antibody modeling with rapid and accurate CDR-H3 loop predictions, making it a powerful tool for biotherapeutics, vaccine development, and large-scale antibody research	[147]
48	2022	Antibody Sequence-Structure Co-Design	RefineGNN (Graph-Based Framework), Iterative Refinement, Coarse-Grained Graph Representation, Feedback Loop Optimization	RefineGNN improves antibody CDR design by co-generating sequence and 3D structure, unlike previous models that assume a fixed structure. It iteratively refines structures while adding residues, ensuring structural relevance and functional integrity	RefineGNN outperforms traditional approaches in sequence quality (perplexity, PPL), structural accuracy (RMSD), and antigen-binding precision (amino acid recovery, AAR). It dynamically refines structures through a feedback loop, ensuring compatibility with fixed framework regions	Researchers developed a generative model that designs CDR sequences and 3D structures simultaneously, treating CDRs as graphs. The model showed superior log-likelihood and effectiveness in designing SARS-CoV-2 neutralizing antibodies, making it a powerful tool for computational antibody design	[250]
49	2022	Antibody Design and Optimization	Diffusion-based generative model, side-chain packing, AMBER force field refinement, equivariant neural networks	Introduces a deep generative model integrating sequence-structure co-design, explicitly conditioning CDR generation on antigen 3D structures. It iteratively refines amino acid types, positions, and orientations to enhance antibody-antigen interactions and optimize existing antibodies	For H1 CDRs, amino acid recovery (AAR) increased from 22.85% (RAbD) to 65.75% (DiffAb), RMSD improved from 2.261 Å to 1.188 Å, and interaction model potential (IMP) increased from 43.88% to 53.63%	Diffusion-based generative model significantly enhances CDR design by improving accuracy, binding affinity, and sequence recovery, making it a powerful tool for therapeutic antibody development	[288]
50	2022	Antibody Binders Prediction and Antibody Generation	CNNs, Generative Adversarial Networks (GANs), Keras, TensorFlow, BLOSUM62 Encoding	Used DL to classify antibody binders and generate synthetic antibodies. CNNs extract features from encoded CDR3 sequences, while GANs create novel CDR3 sequences by learning binder patterns. High-throughput methods combined with deep sequencing enhance classification accuracy	Prediction accuracy: 91.2% (CTLA-4), 92.6% (PD-1). AUC values: 0.90 (CTLA-4), 0.94 (PD-1). Matthews correlation coefficient (MCC) used for robust classification assessment. CNNs trained on BLOSUM62-encoded sequences accurately classify binders	DL improves antibody analysis and optimization. CNNs classify PD-1 and CTLA-4 binders, identify key residues, and GANs generate synthetic antibodies, enhancing efficiency and accuracy in therapeutic antibody discovery	[223]
51	2022	Protein Structure and Sequence Generation	Diffusion Models, AdamW Optimizer, Gradient Accumulation, RosettaFixBB, RosettaRelBB,	This study introduces a generative model for designing proteins with specific 3D structures and chemical properties using Equivariant Denoising Diffusion Probabilistic Models. It integrates sequence, structure, and constraints, outperforming energy-based methods in generating diverse, functional proteins	This model successfully generated biophysically realistic protein sequences, ensuring proper folding and maintaining hydrogen bonding patterns in helices and beta sheets. The generated structures closely matched natural proteins	Equivariant generative models improve protein engineering by learning structural and functional constraints, enabling large-scale, accurate sequence-structure predictions	[327]
52	2022	Protein Modelling, Cell Development	Predictor–Corrector Schemes, Denoising Score Matching (DSM), Conditional SGM and Schrödinger Bridges, Stereographic SGM	Riemannian Score-Based Generative Models (RSGMs) extend Score-Based Generative Models to curved manifolds, improving robotic path planning, geoscience modeling, and protein structure prediction by handling non-Euclidean data distributions effectively	RSGMs demonstrated superior path planning efficiency in robotics and significantly enhanced geoscience modeling accuracy. They provided accurate earthquake and climate event predictions while improving protein structure modeling	RSGMs improve generative modeling for curved data distributions, offering practical applications in robotics, geoscience, and computational biology	[328]
53	2023	Antibody paratope prediction	Graph Neural Network-based tool (Paragraph), Structure modeling via ABodyBuilder and ABlooper	Developed Paragraph, a paratope prediction tool trained on 1,086 antibody-antigen complexes from SAbDab. It is antigen-agnostic, employs simplified feature vectors, and supports vaccine development, antibody design, and high-throughput screening	Paragraph achieved a PR AUC of 0.696, rising to 0.763 for its most confident models. It is 50Ã— faster than Parapred (0.1 s per prediction) and outperforms PECAN. Its simplified feature vector reduces computational complexity while leveraging structural data for improved accuracy	Paragraph is a structure-based tool for paratope prediction that surpasses existing methods by using simpler feature vectors and no antigen information, enhancing prediction accuracy and efficiency	[298]
54	2023	Protein Structures Design	Genie (Diffusion-based generative model), Denoising Diffusion Probabilistic Models (DDPMs), Equivariant neural networks, Triangular multiplicative update layers, Multidimensional scaling (MDS)	Developed Genie, an advanced diffusion-based model for protein structure generation, leveraging equivariant diffusion to learn 3D residue frame distributions, aiding protein engineering, therapeutics, and materials science	Genie generates highly designable, novel, and diverse protein structures, outperforming ProtDiff and RFDiffusion. Its visualization tools enhance structural analysis, and open-source availability promotes further research	Genie advances protein design by exploring novel fold spaces beyond known proteins, improving structural understanding, and enabling efficient generative modeling for cellular engineering and therapeutic applications	[307]
55	2023	Protein Structure and Sequence Design	Protpardelle (Diffusion-based generative model), Self-consistency metrics (scRMSD, scTM-score), MiniMPNN, ProteinMPNN, Clustering algorithms	Developed Protpardelle, an all-atom generative model that co-designs protein structure and sequence, capturing side-chain interactions for realistic configurations while ensuring functional integrity	Protpardelle generates high-quality and diverse all-atom protein structures, validated using self-consistency metrics, chemical quality evaluations, and secondary structure analysis. It avoids energy function relaxation to maintain accuracy	Protpardelle advances protein engineering by generating realistic protein structures and sequences, capturing natural features without relying on predefined backbones or rotamers	[300]
56	2023	Full-Atom Generation of Antibodies	AbDiffuser (Diffusion model with physics-informed priors), Graph Neural Networks (E(n) Equivariant GNNs, FA-GNN), Aligned Protein Mixer (APMixer), High-throughput screening, Sequence transformers (BERT)	Developed AbDiffuser, a physics-informed diffusion model for generating accurate and efficient full-atom antibody structures and sequences, optimizing therapeutic applications	AbDiffuser achieved high precision in antibody structure and sequence generation. In vitro testing on HER2 binders showed 57.1% strong binding rates, with top candidates performing comparably to Trastuzumab	AbDiffuser integrates diffusion models, physics-based priors, and memory-efficient architectures to advance computational antibody design, improving precision, structural accuracy, and therapeutic potential	[290]
57	2023	Antibody Optimization using AbGAN-LMG	AbGAN-LMG, AbGAN-ESM2–150 M, AbGAN-BERT2DAb, AbGAN-AntiBERTy, IgFold, RGN2, DRN-1D2D_Inter, AbGAN-FEGS, AbGAN-No-Guided, ProteinGAN	AbGAN-LMG, a GAN-language model hybrid, optimizes antibody sequences, improving binding affinity and developability, demonstrating superior performance	Generated antibodies demonstrated higher affinity and better developability, with a 70% improvement over traditional optimization methods	AbGAN-LMG enhances antibody sequence design, making AI-assisted therapeutics more efficient and precise	[85]
58	2023	Estimation of Data Distribution Gradients with Langevin Dynamics	Annealed Langevin dynamics, Standard Langevin dynamics	Langevin dynamics is applied for estimating data gradients, preventing distribution collapse, achieving state-of-the-art results on generative benchmarks	Inception Score of 8.87, FID of 25.32, surpassing GANs and conventional score-matching models	Langevin dynamics stabilizes generative modeling, improving sample diversity and generative efficiency	[329]
59	2023	Protein Engineering using UniRep	Average Linkage (Euclidean Distance), Average Linkage (Levenshtein Distance), Levenshtein Algorithm, mLSTM (multi-layer Long Short-Term Memory), Softmax Regression	UniRep integrates DL and Gaussian processes to improve protein engineering by learning statistical representations of unlabeled amino acid sequences	Outperforms Rosetta in stability prediction, achieving a Spearman’s correlation of 0.59 vs. 0.42	UniRep streamlines protein engineering, demonstrating state-of-the-art efficiency in protein stability predictions	[304]
60	2023	Ligand Binding Site Prediction	Fpocket, DeepSite, Kalasanty, DeepSurf, GAT, GCN, GCN2, SchNet, DimeNet + + , EGNN	EquiPocket improves binding site prediction by using graph-based representations, preserving protein structures without voxelization distortion. It captures detailed surface geometry and integrates chemical and spatial structures, enhancing predictive accuracy	EquiPocket was benchmarked on multiple protein datasets, including scPDB (17,594 structures), COACH420 (2,123 proteins), HOLO4K (4,000 proteins), and PDBbind (3,104 proteins). Performance metrics like DCC (Distance of Closest Contact) and DCA (Distance-based Contact Area) demonstrated superior results compared to CNN-based methods	EquiPocket, an E(3)-equivariant Graph Neural Network, predicts protein binding sites with high accuracy and robustness. Its superior performance in benchmarks highlights its potential as a valuable tool for drug discovery	[249]
61	2023	Antibody Structure Prediction	AlphaFold-Multimer, AlphaFold2, EquiFold, ABodyBuilder, ABlooper, IgFold, RAdam, Cosine annealing scheduler	ImmuneBuilder predicts immune protein structures, including antibodies and nanobodies, using DL. It features specialized models like ABodyBuilder2, NanoBodyBuilder2, and TCRBuilder2	ABodyBuilder2 achieved an RMSD of 2.81 Å for CDR-H3 loops, outperforming AlphaFold2. The tool provides error estimates and a web server for accessibility	ImmuneBuilder is a highly efficient, accurate tool for antibody modeling, improving prediction speed and accuracy for biotherapeutic applications	[291]
62	2023	Antibody Structure Prediction	IgFold, AlphaFold, AlphaFold-Multimer, DeepAb, ABlooper, MMseqs2, and MODELLER	IgFold predicts antibody structures from sequences using graph networks and DL. It enhances hypervariable loop modeling and significantly expands the known structural antibody space	IgFold achieved a median RMSD of 1.95 Å for CDR-H3 loops and improved large-scale structural analysis by 500-fold	A rapid, AI-driven antibody structure prediction model that matches or outperforms AlphaFold, expanding antibody modeling capabilities	[24]
63	2023	Immunoglobulin Language Model, antibody sequence generation	ANARCI, Rosetta, CamSol-Intrinsic, BioPhi, IgLM (Infilling by Language Modeling), GPT-2 Transformer, and ProGen2	This study addresses antibody developability issues by generating full-length antibody sequences with enhanced stability and reduced aggregation. IgLM is a transformer-based model trained on 558 million sequences, optimizing antibody discovery by controlling sequence modifications while maintaining structural integrity	IgLM enables targeted sequence modifications while preserving antibody integrity. It achieved high developability scores, improving CDR loop libraries and therapeutic relevance. Comparative analyses confirmed its superior stability over ProGen2-OAS	IgLM, trained on 558 million sequences, significantly enhances antibody optimization by addressing solubility, aggregation, and immunogenicity challenges, streamlining therapeutic development	[215]
64	2023	Protein Backbone Modelling in 3D	SMCDiff, DDPM (Denoising Diffusion Probabilistic Model), Particle Filtering Algorithm	SMCDiff enables conditional motif-scaffolding, generating longer, diverse scaffolds (up to 80 residues) while maintaining AlphaFold2 accuracy. It overcomes previous limitations of short scaffolds and inefficient generation, advancing vaccine and enzyme design	SMCDiff generates scaffolds up to 80 residues long, significantly extending previous limits (20 residues). Empirical results confirmed its efficiency and structural diversity, making it a valuable tool for protein engineering	SMCDiff efficiently samples scaffold structures, ensuring design flexibility and accuracy, making it a significant advancement for motif-scaffolding applications	[293]
65	2024	Antibody Design	ZeRoShot, generative AI models, grid search, Principal Component Analysis (PCA), support vector machine (SVM)	A generative AI model was used to design HER2-targeting antibodies de novo, eliminating the need for optimization. It generated epitope-specific binders, reducing reliance on traditional screening or immunization	High-throughput screening identified potential binders, with SPR analysis confirming low nanomolar affinity. 71 antibodies showed high binding efficiency, with 11 matching trastuzumab potency	The AI-driven model significantly accelerates therapeutic antibody discovery, achieving high binding success and eliminating the need for extensive optimization	[142]
66	2024	CDR Design	Equivariant Graph Neural Networks, Data Augmentation Strategy, LSTM-based Deep Generative Models	The AbFlex model enhances CDR design using an equivariant graph neural network (EGNN) and CDR augmentation strategies. It improves generalization across numbering schemes, refining antigen binding	Benchmarking results showed an RMSD of 1.568 Å and an AAR of 37.54%, with 80% of designed antibodies exhibiting improved binding energies over wild types	AbFlex optimizes CDR sequences with structural accuracy, improving binding affinity and robustness across different antibody designs	[289]
67	2024	Foundation model, natural and chemical language	T5 Model, Multi-task Learning, Fine-tuning Techniques, NeMo Toolkit, Data Mixing Strategy	This study introduces nach0, an LLM designed for biomedical problem-solving, molecular recognition, synthesis, and chemical property prediction. Nach0 integrates linguistic and chemical knowledge, excelling in biomedical question answering, molecular generation, synthesis planning, and reaction prediction. Trained using the NeMo framework, it offers robust multi-task performance	Nach0 outperformed SciFive and FLAN in molecular tasks, achieving an F1 score of 82.24%. It excelled in multitasking and generated viable JAK3 inhibitors in 45 min compared to Chemistry42’s 72-h runtime	Nach0, a multi-domain LLM, outperforms leading models in scientific literature and molecular task generation, proving effective in biomedical and chemical research applications	[309]
68	2024	Protein structure generation	RFDiffusion, P-SEA, Autoregressive (AR) model, Randomized sampling approach, 31-dimensional Gauss integrals	This study developed FoldingDiff, a diffusion-based model for generating diverse, foldable protein structures. It iteratively refines random conformations into stable structures using angle-based representation, ensuring accurate geometric properties. The model closely matches experimental structures and enables biologically plausible protein design	FoldingDiff generated 177 viable protein backbones out of 780, achieving a self-consistency TM score of ≥ 0.5. Clustering analysis confirmed structural diversity, while Ramachandran plots validated correct folding properties	FoldingDiff simulates natural protein folding, producing viable and diverse structures with validated experimental consistency. It advances computational protein design and structural biology research	[330]
69	2024	Antibody design	IgDiff (Diffusion-based generative model), Deep probabilistic models, Structural consistency validation	Developed IgDiff, a diffusion-based generative framework for designing novel, highly designable antibody structures, particularly optimizing CDRs for enhanced binding. Unlike slow physics-based methods, IgDiff leverages large datasets for rapid and efficient predictions	IgDiff-designed antibodies were experimentally validated, showing high expression yields. It maintained realistic backbone dihedral angles and achieved 93.3% structural self-consistency in light chains. It outperformed RFDiffusion in designing CDR loops and pairing light/heavy chains, demonstrating superior designability and functionality	IgDiff significantly advances computational antibody design by generating highly designable, experimentally viable antibodies with superior CDR loop modeling and structural accuracy, making it a powerful tool for therapeutic development	[294]

## Challenges and Future Prospects of Antibody Designing

Antibody design faces several challenges, including predictive limitations, computational complexity, data scarcity, and time-intensive processes [35, 37, 38, 331]. Many key physicochemical properties, such as stability, solubility, immunogenicity, and affinity, are challenging to predict solely from sequences and often require experimental validation for confirmation [35, 331]. These properties directly impact efficacy, manufacturability, and patient safety [10, 11, 331]. AI has contributed to advancements in antibody design by improving predictive accuracy, assisting in CDR sequence generation, enhancing homology modeling, and optimizing antibody-antigen interactions [22, 35, 37, 231, 306]. Despite ongoing advancements, AI faces challenges in generalization, dataset limitations, and computational demands, which may affect the seamless translation of in silico designs into real-world therapeutics. However, ongoing improvements in training methodologies and data availability have the potential to enhance its predictive power and applicability [22]. The choice of a machine learning algorithm depends on dataset availability and application objectives. DL typically benefits from large datasets, but for smaller datasets, traditional ML methods may sometimes perform better unless transfer learning or data augmentation techniques are applied [11]. Overcoming these obstacles requires integrating AI with physics-based modeling, multi-objective optimization, and experimental feedback loops to enhance predictive power, manufacturability, and patient safety [22, 184, 290, 294, 332].

### Stability challenges

The stability of an antibody is critical for therapeutic efficacy, safety, and manufacturability, directly influencing shelf life, formulation, and patient compliance [13]. However, predicting stability remains a challenge due to the complex interplay between sequence, structure, and environmental conditions, despite advancements in computational modeling [13]. While the primary sequence provides valuable insights, it cannot fully determine stability, as subtle structural variations and intermolecular interactions significantly impact behavior [331]. Computational methods face limitations in accurately predicting these variations, making experimental validation an essential complement. Key challenges include thermal instability, leading to denaturation and efficacy loss; chemical degradation, such as oxidation and deamidation, which affect safety and require extensive testing; and aggregation, influenced by factors like pH and concentration, which can hinder therapeutic function and trigger immune responses [13, 89, 107, 138, 278, 331, 333]. Conformational stability is important for maintaining antibody functionality and reducing the risk of immunogenicity or aggregation [10, 331]. Environmental factors such as temperature, pH, and ionic strength can trigger denaturation, while protein misfolding and intermolecular interactions further impact stability [331]. Post-translational modifications, such as glycosylation, may enhance or destabilize antibody structure [10].

Long-term stability challenges, including temperature fluctuations and light exposure, often necessitate optimization strategies such as lyophilization to improve efficacy and manufacturability [331]. Thus, while predictive models have limitations, experimental validation remains an important tool for ensuring stability assessments [331]. AI-based stability prediction methods face challenges in fully capturing stability variations across different pH, temperature, and formulation conditions [334]. Trade-offs between stability, affinity, and immunogenicity add complexity to multi-objective optimization, and current AI models have limitations in fully capturing these intricate relationships [38]. Addressing these challenges requires integrating AI with experimental validation and optimization techniques to enhance predictive accuracy and develop stable, effective therapeutics.

### Immunogenicity challenges

Immunogenicity is an important consideration in antibody development, influencing efficacy, safety, and regulatory approval [13, 212]. Highly immunogenic antibodies can trigger immune responses, neutralizing their therapeutic effects and increasing the risk of adverse reactions, including hypersensitivity and autoimmunity [13, 98, 212]. AI models face data limitations, as well-annotated datasets of immunogenic and non-immunogenic antibodies remain limited [98, 212, 335]. Additionally, immune system interactions are highly variable, influenced by host genetics, post-translational modifications, and prior exposures, making sequence-based immunogenicity predictions challenging and context-dependent [11, 13, 212]. AI models have limitations in accurately predicting T-cell and B-cell epitopes, which are key determinants of immune reactions [336]. AI-generated antibodies may exhibit low immunogenicity, but without proper constraints, they could deviate from natural antibody structures, potentially affecting expression or functionality [337]. Addressing these challenges requires integrating AI-driven predictions with experimental validation to improve the reliability of immunogenicity assessments.

### Solubility challenges

Solubility is essential for antibody stability, efficacy, and manufacturability. Poor solubility can lead to aggregation, reducing function and increasing immunogenicity risks [89, 96, 98, 99]. AI-based solubility prediction faces challenges due to limited high-quality training data and the complexity of solubility-influencing factors such as amino acid composition, surface charge, and hydrophobicity [22, 97, 99, 338]. AI-generated antibodies may include aggregation-prone regions, which could impact manufacturability and therapeutic efficacy if not properly addressed [339]. The design process, even with advanced computational methods, may not always capture the full complexity of amino acid interactions that contribute to protein stability [96, 97, 340]. These aggregation-prone regions can lead to increased aggregation rates, potentially affecting solubility, functional activity, and immunogenicity risk [103, 341]. Enhancing solubility may require mutations that alter charge distribution and hydrophobicity, which could impact affinity or stability, adding complexity to multi-objective optimization [89, 96, 97, 108, 342]. Addressing these challenges requires a balance between computational predictions and experimental validation to develop stable and therapeutically effective antibodies.

### Affinity challenges

Antibody affinity is essential for therapeutic efficacy, dose optimization, and stability. High-affinity antibodies improve pathogen neutralization and therapeutic targeting, allowing for lower doses and reducing side effects [162, 205, 264]. However, optimizing affinity remains challenging due to the complexity of antibody-antigen interactions, which involve structural conformations, thermodynamics, and binding kinetics [10, 83, 161, 162, 205, 264, 331]. AI models face challenges due to the limited availability of high-quality structural data and often rely on small, curated datasets, which may introduce biases [169]. Traditional affinity maturation techniques, such as phage display and hybridoma methods, are costly and time-consuming, while AI-generated predictions still require experimental validation to confirm accuracy and functional performance [98]. The vast combinatorial space of CDR sequences adds complexity to optimization, requiring AI to balance high-affinity binder selection with the maintenance of other essential properties. Addressing these challenges involves integrating AI-driven predictions with experimental feedback to improve affinity maturation strategies efficiently.

#### Broader challenges in AI-based antibody design

AI-based antibody design faces broader challenges beyond optimizing individual properties, which can affect its generalizability in real-world applications. AI models can face challenges with generalization, sometimes generating variations of known antibodies rather than entirely novel candidates, which may affect adaptability to emerging diseases [11, 22, 26, 34]. Overfitting remains a challenge in AI-driven antibody design, potentially limiting the exploration of diverse solutions and requiring advanced regularization techniques to enhance innovation [193].

AI-generated sequences may exhibit limited conformational flexibility in some cases, potentially affecting affinity, accuracy, and functionality, especially when models rely heavily on predefined templates and training data patterns [22]. A key challenge is the limited availability of high-quality data, which can impact AI’s ability to generalize across diverse therapeutic targets. AI models require diverse, unbiased datasets, but data scarcity—particularly for rare or newly emerging pathogens—can impact model accuracy and generalizability [210, 211, 218, 343, 344]. Integrating in vitro and in vivo experimental data remains challenging due to biological variability, which can affect AI’s ability to generalize across different biological systems. Navigating the vast sequence and chemical space of antibodies adds another layer of complexity [345].

Binding affinity and specificity predictions require advanced learning frameworks to model intricate molecular interactions [22, 88, 342]. Many-to-many binding dynamics introduce nonlinear dependencies that traditional models may struggle to capture, making optimization more difficult [346, 347]. Parameter interdependence further complicates AI-driven design, as improving one property—such as affinity—may negatively impact stability, pharmacokinetics, or immunogenicity. Computational inefficiencies remain a challenge in AI-based antibody optimization. Traditional sampling algorithms and statistical energy functions may struggle to efficiently explore the vast search space, sometimes getting trapped in local optima [11, 26, 34, 158, 288]. Antibody structural complexity further complicates predictive modeling, as capturing 3D conformational dynamics and their effects on binding interactions requires high computational resources. Designing 3D ligands, CDRs, and accurately modeling protein interactions remains a challenge, though advances in AI-driven structural modeling are improving predictive accuracy [344, 348].

Integrating multi-omics data improves predictive accuracy by combining genomic, proteomic, and transcriptomic insights but adds computational complexity due to format and context variations. Antibody optimization requires balancing efficacy, safety, and manufacturability, making multi-objective parameter refinement challenging [349, 350]. Optimizing different antibody regions simultaneously demands robust algorithms to navigate trade-offs between binding strength, stability, and immunogenicity [349–351]. Overcoming these challenges requires advancements in AI methodologies, improved data curation, and better integration with experimental approaches. Enhancing AI with physics-based modeling, reinforcement learning, and deep generative models will improve antibody discovery, making AI-driven design more effective for next-generation therapeutics.

### Future prospects

The validation of AI-generated antibodies presents significant challenges due to the complexity of biological systems, necessitating extensive in vitro and in vivo testing [35]. These processes are time-consuming and resource-intensive, requiring specialized equipment, skilled personnel, and high costs, which further slow development. Iterative feedback loops extend timelines as AI models refine predictions based on experimental results. The limited predictive power of current models and discrepancies in antibody-antigen interactions necessitate further improvements. Additionally, regulatory hurdles add complexity, delaying approval processes [22].

Overfitting remains a challenge, as some AI models generate candidates that resemble known antibodies rather than entirely novel structures [85, 290]. High-throughput experimental data can mitigate overfitting by exposing AI models to greater sequence diversity, promoting the creation of novel yet functional antibodies [142]. In some cases, AI-generated antibodies may exhibit limited structural flexibility, potentially affecting binding efficacy, emphasizing the need for experimental validation to refine AI predictions. Integrating ligand- and structure-based design strategies may also enhance predictive accuracy by capturing atomic-level interactions and improving AI-driven antibody discovery reliability [352].

While sequence-based DL methods have successfully generated antibody candidates, some models face challenges in achieving precise antigen specificity due to their limited ability to model atomic-level interactions [19, 163, 257, 260]. This limitation hinders precise antibody design, as these models primarily rely on sequence patterns rather than fully capturing structural and functional constraints. While tools like AlphaFold2 effectively predict per-amino-acid orientations, they face challenges in generating diverse and functionally relevant protein structures [28]. Deep generative diffusion models show promise in addressing these challenges by facilitating the design of CDR structures, novel sequences, and molecular 3D conformations [178, 288, 294, 328, 353, 354]. These models contribute to bridging the gap by translating sequence and multiple sequence alignments (MSAs) into atomic-level 3D structures, potentially enhancing precision for specific antibody targets [293, 327, 330]. AI-driven platforms that combine deep sequencing-based computational methods with advanced data processing further expand antibody exploration. While these advancements show promise, experimental validation remains essential to ensure real-world applicability and functional reliability.

To further improve AI-driven antibody design, several key strategies are needed. Physics-based modeling, including molecular dynamics simulations and free energy calculations, may provide additional mechanistic insights into antibody-antigen interactions, complementing AI-driven sequence and structure predictions. Multi-objective optimization is critical for balancing stability, affinity, immunogenicity, and manufacturability, with reinforcement learning and evolutionary algorithms optimizing multiple properties simultaneously [349, 350]. Additionally, improving data integration and augmentation is necessary, as expanding high-quality, experimentally validated datasets and leveraging deep sequencing-driven computational techniques will enhance model robustness [210, 211, 218]. Data augmentation strategies, such as self-supervised learning and generative modeling, can help overcome data scarcity and improve AI generalization. Explainable AI (XAI) is expected to play a key role in increasing trust in AI-generated predictions by enhancing transparency in decision-making for scientists and regulatory authorities. As AI continues to evolve, integrating advanced computational methods with experimental validation will be crucial for addressing current limitations and advancing next-generation antibody therapeutics and precision medicine.

## Conclusion

This review highlights the transformative impact of AI in antibody drug design, with deep generative models demonstrating significant potential in complementing traditional methods by automating CDR sequence generation, refining antibody-antigen interaction modeling, and improving predictive accuracy. These advances streamline sequence optimization and accelerate drug development. Despite these advancements, challenges persist, including data quality limitations, model interpretability, high computational costs, and the vast complexity of antibody sequence space. Addressing these issues requires integrating ligand- and structure-based design strategies, incorporating explainable AI (XAI) for transparency, and continuously improving data availability and model generalization. Future AI-driven protein design is likely to focus on scaling models and datasets to improve generative performance, with the aim of enabling more precise and function-specific antibody designs. The integration of AI with natural language models has the potential to simplify design workflows, while laboratory automation may aid in validation and real-world applications. However, AI-driven antibody design faces challenges in generalization, real-world applicability, and reliance on high-quality experimental data. Despite these challenges, AI-driven approaches—particularly in oncology—offer promising potential for automated de novo antibody design and optimization. Startups such as Atomwise and BenevolentAI are contributing to advancements in AI-driven drug discovery by exploring faster and potentially more cost-effective solutions. With continued advancements in deep learning, physics-based modeling, and experimental validation, AI is likely to play an increasingly important role in accelerating therapeutic antibody development.

## Data Availability

No datasets were generated or analysed during the current study.

## Abbreviations

3D: 3 Dimensional
ADCs: Antibody-Drug Conjugates
ADMET: Absorption, Distribution, Metabolism, Excretion and Toxicity
AI: Artificial Intelligence
AR: Autoregressive Model
AUC: Area Under the Curve
bsAbs: Bispecific Antibodies
CAR: Chimeric Antigen Receptor
CDR: Complementarity-Determining Region
CM: Co-stimulatory molecule
CNNs: Convolutional Neural Networks
CTLA-4: Cytotoxic T-Lymphocyte Associated Protein 4
DGMs: Deep Generative Models
DL: Deep Learning
DNN: Deep Neural Networks
EGFR: Epidermal Growth Factor Receptor
Fab: Fragment antigen-binding
Fc: Fragment crystallizable
FDA: Food and Drug Administration
GAN: Generative Adversarial Networks
GENTRL: Generative Tensorial Reinforcement Learning
GPU: Graphics Processing Unit
HC: Heavy Chain
LC: Light Chain
LLM: Large Language Models
LSTM: Long short-term memory
mAbs: Monoclonal Antibodies
MET: Mesenchymal-Epithelial Transition Factor
ML: Machine Learning
NFAT: Nuclear Factor of the Activated T cell
NGS: Next Generation Sequencing
NK: Natural Killer
NSCLC: Non-Small Cell Lung Cancer
OAS: Observed Antibody Space
PD-1: Programmed Cell Death Protein1
PDB: Protein Data Bank
PGFS: Policy Gradient for Forward Synthesis
PSSM: Position-Specific Scoring Matrix
RL: Reinforcement Learning
RNNs: Recurrent Neural Network
ROC: Receiver-Operating Characteristic
SASA: Solvent Accessible Surface Area
scFv: Single-Chain Variable Fragment
SMILES: Simplified Molecular Input Line Entry System
SQL: Structured Q-learning
TL: Transfer Learning
VAE: Variational Autoencoders
VAMP: Variable Allocation Markov Decision Process
VEGF: Vascular Endothelial Growth Factor
VH: Heavy Chain Variable
VL: Light Chain Variable
XAI: Explainable AI
